# Recent Advances in Drug Discovery for Triple-Negative Breast Cancer Treatment

**DOI:** 10.3390/molecules28227513

**Published:** 2023-11-09

**Authors:** Domiziana Masci, Chiara Naro, Michela Puxeddu, Andrea Urbani, Claudio Sette, Giuseppe La Regina, Romano Silvestri

**Affiliations:** 1 Department of Basic Biotechnological Sciences, Intensivological and Perioperative Clinics, Catholic University of the Sacred Heart, Largo Francesco Vito 1, 00168 Rome, Italy; domiziana.masci@unicatt.it (D.M.); andrea.urbani@unicatt.it (A.U.); 2 Department of Neurosciences, Section of Human Anatomy, Catholic University of the Sacred Heart, Largo Francesco Vito 1, 00168 Rome, Italy; chiara.naro@unicatt.it (C.N.); claudio.sette@unicatt.it (C.S.); 3 GSTeP-Organoids Research Core Facility, Fondazione Policlinico Universitario A. Gemelli, IRCCS, 00168 Rome, Italy; 4 Laboratory Affiliated to Istituto Pasteur Italia—Fondazione Cenci Bolognetti, Department of Drug Chemistry and Technologies, Sapienza University of Rome, Piazzale Aldo Moro 5, 00185 Rome, Italy; michela.puxeddu@uniroma1.it (M.P.); giuseppe.laregina@uniroma1.it (G.L.R.)

**Keywords:** triple-negative breast cancer, cancer, inhibitors, drug resistance, PARP, kinases, microtubules, antibody drug conjugates, combination therapy

## Abstract

Triple-negative breast cancer (TNBC) is one of the most heterogeneous and aggressive breast cancer subtypes with a high risk of death on recurrence. To date, TNBC is very difficult to treat due to the lack of an effective targeted therapy. However, recent advances in the molecular characterization of TNBC are encouraging the development of novel drugs and therapeutic combinations for its therapeutic management. In the present review, we will provide an overview of the currently available standard therapies and new emerging therapeutic strategies against TNBC, highlighting the promises that newly developed small molecules, repositioned drugs, and combination therapies have of improving treatment efficacy against these tumors.

## 1. Introduction

Breast cancer (BC) is a serious worldwide disease that threatens women’s health. Globally, 2.3 million new cases of breast cancer have been diagnosed in 2020, with 685,000 estimated deaths. 

Triple-negative breast cancer (TNBC) accounts for 15–20% of all invasive breast cancer and is considered as one of the most heterogenous and aggressive breast cancer subtypes. In fact, it is characterized by a high level of cell invasiveness and a tendency to metastasize to various organs, frequently affecting the brain, lungs, and liver, with an average survival time of 18 months.

Notable ethnic and racial disparities in TNBC incidence have been reported so far. African American women have a higher risk of developing TNBC compared to other racial and ethnic groups. Indeed, most of the available evidence shows that black race, younger age (34% were 18–29 years of age), and later tumor stage are associated with the TN subtype compared to other breast cancer subtypes and suggest a positive correlation between increased parity (having more children) and the risk of developing TNBC, while indicating a negative association between the duration of breastfeeding and the risk of developing this disease. Additionally, excess weight represents a factor significantly contributing to TNBC occurrence. In this respect, Trivers et al. demonstrated in their case study that overweight or obese [odds ratio (OR) = 1.89, 95% confidence interval (CI): 1.22–2.92] women were at a higher risk of developing TNBC [1]. In a different study that analyzed clinicopathological data from 112 triple-negative breast cancer patients in a Turkish hospital over a 5-year period, it was reported that, at the time of diagnosis, 30 patients (26.8%) were normal weight or underweight, while 82 (73.2%) were classified as overweight/obese [2]. Although a potential association exists between diabetes and obesity outcomes, the available information on how diabetes may affect the incidence of TNBC is limited. 

Furthermore, there is an indication of a potential influence of diet on the risk of developing TNBC. This is based on epidemiological studies that have demonstrated a slightly significant inverse relationship between a high intake of total vegetables (with a relative risk of 0.82 and a 95% confidence interval of 0.74–0.90) [3] or a diet rich in fruits and vegetables (with a relative risk of 0.85 and a 95% confidence interval of 0.76–0.95) [4] and the risk of developing estrogen receptor-negative (ER-negative) breast cancer.

These findings highlight the link between lifestyle behaviors, race, and the probability of developing TNBC. In fact, women, especially of African ancestry, living in areas of low socioeconomic status were more likely to be diagnosed with TNBC.

Triple-negative breast cancer is characterized by the absence of expression of the estrogen receptor (ER), progesterone receptor (PR), and human epidermal growth factor receptor-2 (HER2); therefore, it will not respond to hormonal therapy or medicines that target HER2, rendering targeted therapeutic options limited [5,6]. Triple-negative breast cancers are highly heterogenous tumors and studies from the Lehmann group, based on genomic and transcriptomic analysis, have led to the identification of six distinct and stable molecular subtypes: basal-like 1 (BL1), basal-like 2 (BL2), immunomodulatory (IM), mesenchymal (M), mesenchymal stem-like (MSL), and luminal androgen receptor (LAR) [5,7]. 

The primary obstacles in the conventional therapeutic approach to TNBC lie in dealing with intrinsic and acquired chemoresistance. Given the considerable diversity within the TNBC subtypes, multiple lines of evidence suggest that the implementation of molecular stratification for TNBC patients in clinical practice could be a key step in addressing intrinsic chemoresistance effectively. As a proof of concept, these subtypes were replicated in breast cancer cell lines and subsequently used to guide the selection of pharmacological treatments. The BL1 subtype exhibited abnormal levels of gene expression (MYC, PIK3CA, CDK6, AKT2, KRAS, FGFR1, IGF1R, CCNE1, CDKN2A/B, BRCA2, PTEN, MDM2, RB1, and TP53) related to cell proliferation, cell cycle regulation, and DNA repair. Therefore, it is generally considered one of the more aggressive subgroups, with a higher risk of recurrence and metastasis. Santonja et al. demonstrated that BL1 cell lines appeared to be particularly sensitive to chemotherapy regimens including platinum agents [8]. 

The BL2 subtype has a unique gene expression profile characterized by the upregulation of genes associated with growth factor signaling pathways (EGFR, MET, NGF, Wnt/β-catenin, and IGF-1R) and myoepithelial markers. As well as BL1, BL2 is associated with early recurrence and metastasis. The pathological response (pCR) rate for patients with the BL2 subtype, after neoadjuvant chemotherapy, is reported to be only 10% due to the poor sensitivity to the combination regimen [9]. 

The IM subtype is composed of immune antigens and genes involved in cytokine and core immune signal transduction pathways. Patients with tumors categorized as the IM subtype tend to have a more favorable prognosis compared to other TNBC subtypes. An examination of the gene expression data within the IM subtype and the identification of transcripts associated with lymphocytes suggest that these IM tumor samples may harbor tumor-infiltrating lymphocytes (TILs). Recent findings from two adjuvant phase III trials have demonstrated a favorable outcome in TNBC patients with higher levels of TILs in their tumors [10]. In addition, given the immune-rich nature of the IM subtype, there is growing interest in exploring immunotherapeutic approaches as a treatment option for these patients.

The M subtype is characterized by a highly activated cell migration-related signaling pathway (regulated by actin), interaction between extracellular matrix and cell receptor, and differentiation pathways (Wnt pathway, anaplastic lymphoma kinase pathway, and transforming growth factor (TGF)-β signaling). Due to these distinctive features, it is often referred to as metaplastic breast cancer. The M subtype exhibits tissue characteristics resembling sarcoma or squamous epithelial cells and tends to develop resistance to chemotherapy. As a result, patients with the M subtype may be considered for treatment with the mammalian target of rapamycin and phosphatidylinositol-3-kinase inhibitors or drugs that target the process of epithelial–mesenchymal transition (EMT) [9]. 

Both M and MSL share an elevated expression of genes involved in EMT and growth factor pathways; however, the MSL subtype expresses low levels of cell proliferation-related genes and high levels of stemness-related genes, HOX genes, and mesenchymal stem cell-specific markers [9]. MSL subtype patients can be treated with the multifamily tyrosine kinase inhibitor dasatinib that binds to the kinase domain of Bcr-Abl.

The LAR subtype exhibits a notably distinct gene expression profile compared to other TNBC subtypes. Despite the absence of expression of ER, it is characterized by highly activated hormone-related signaling pathways (including steroid synthesis, porphyrin metabolism, and androgen/estrogen metabolism). Notably, the androgen receptor (AR) is highly expressed in LAR subtype breast cancer, with its mRNA level being approximately nine times greater than in other TNBC subtypes [11]. Therefore, anti-AR therapy (e.g., bicalutamide as an AR antagonist) is recommended for patients with LAR subtype breast cancer.

Compared to non-TNBC patients, TNBC patients suffer from worse clinical outcomes, largely due to heterogeneity, lack of treatment options, and frequency of recurrence and metastasis [12,13,14]. Women diagnosed with TNBC between 2012 and 2018 showed a 5-year overall survival rate of 77%, compared with 93% in breast cancer patients of other types [15]. Recently, the main treatment methods for TNBC are mastectomy or breast-conserving surgery, radiation therapy, and chemotherapy. Due to its molecular heterogeneity, poor cell differentiation, high malignancy, lack of molecular targets, and poor prognosis often ascribed to resistance to chemotherapeutic agents, there is no targeted therapy to prevent the recurrence of TNBC [16]. Overall standard neoadjuvant chemotherapy yields a pathological complete response in a limited proportion of patients with TNBC (35–45% in 2020) [17]. Hence, continuous efforts are being pursued to develop more targeted therapies, particularly for patients with advanced TNBC who poorly respond to current chemotherapeutic agents and, even after a good response, show rapid disease progression.

Thus far, TNBC is very difficult to treat. Several novel agents, such as single-target drugs and repurposing drugs, have been developed and evaluated in clinical studies for immuno- and targeted-TNBC therapies. In this review, we will focus on the currently available standard therapies against TNBC and on the new advancement in the development of small molecules for TNBC.

## 2. Conventional Chemotherapy

Conventional chemotherapy, consisting of different combinations of anthracycline, taxane, cyclophosphamide, and fluorouracil, is the mainstay of adjuvant systemic treatment for most patients with early-stage TNBC, although sometimes schedules are limited by toxicity and tumor response. At present, the 90% of drug failures in metastatic cancers is caused by the development of multidrug resistance by tumor cells. In this respect, tumor cells have given rise to the ability to survive after chemotherapeutic exposure through the employment of several mechanisms, such as APC transporters, β-tubulin III, mutations in DNA repair enzymes such as topoisomerase II and DNA mismatch repair enzymes, alterations in genes involved in apoptosis, ALDH1 and glutathione (GSH)/Glutathione-S-transferase (GST), and NF-kB signaling pathways [18].

In addition to standard chemotherapy, the addition of any other drugs has been regarded as a new regimen [19].

**Figure 1:**
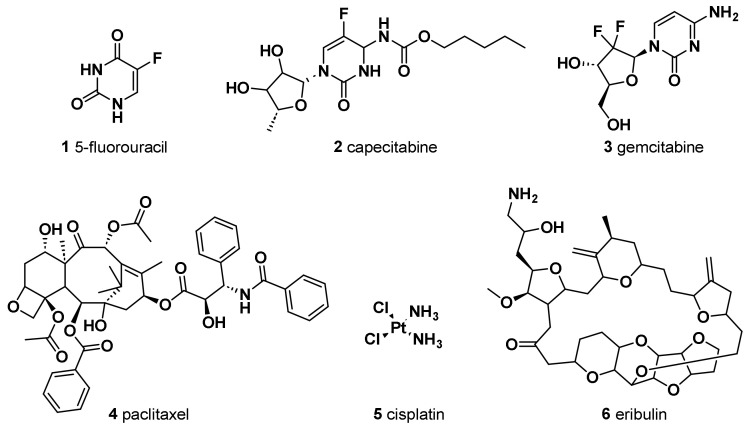
Chemical structures of conventional chemotherapeutic agents.

The antimetabolite 5-fluorouracil (5-FU) (**1**) (Figure 1) is widely employed in treating different cancers, including breast cancer. The chemical structure of this antitumor agent is comprised of a heterocyclic aromatic ring with high similarity to uracil, bearing a fluorine atom in 5C position of the aromatic ring. Continuous progress in comprehending its mechanism of action has led to its extensive anticancer applications [20]. It has been indicated that the anticancer effect of 5-FU, when it is administered as a single agent chemotherapeutic drug, is mainly induced by its conversion to three active metabolites: fluorodeoxyuridine monophosphate (FdUMP), fluorodeoxyuridine triphosphate (FdUTP), and fluorouridine triphosphate (FUTP). In fact, these metabolites cause cell injury by two different mechanisms. Firstly, the binding of FdUMP to the nucleotide binding site of thymidylate synthase (TS), an essential enzyme for catalyzing the reductive methylation of deoxyuridine monophosphate (dUMP) to deoxythymidine monophosphate (dTMP), with the folate cofactor 5,10-methylene tetrahydrofolate (CH_2_THF), as the methyl donor, resulted in the formation of a stable ternary TS–FdUMP–CH_2_THF complex blocking access of dUMP to the nucleotide binding site and, therefore, in the inhibition of dTMP synthesis. Since dTMP is essential for DNA replication and repair, its depletion causes lethal DNA damage. Furthermore, the cytotoxic effect of 5-FU is also triggered by the incorporation of its active metabolite, FUTP, into RNA. This not only resulted in the inhibition of the processing of pre-rRNA into mature rRNA but also interfered with the post-transcriptional modification of tRNAs and the assembly and activity of snRNA/protein complexes, thereby inhibiting splicing of pre-mRNA. Therefore, the misincorporation of 5-FU has the potential to disrupt various facets of RNA processing, leading to significant effects on cellular metabolism and survival.

Moreover, the outbreak of drug resistance, due to the upregulation of ATP-binding cassette (ABC) transporters, is a very common phenomenon among breast cancer patients who were administered 5-FU. In fact, it has been demonstrated that multidrug-resistant protein-1 (ABCC1/MRP1), breast cancer resistance protein (ABCG2/BCRP), and multidrug-resistant protein-8 (ABCC11/MRP8), expressed more frequently in TNBC compared to other breast cancer subtypes, are responsible for the upregulation of ABC transporters that utilize ATP to efflux various compounds—including a wide range of anticancer drugs—across cellular membranes for conferring resistance to 5-FU and other drugs that represent the backbone of current TNBC treatment. The biological effects of developing 5-FU resistance include a decline in apoptosis, disorder in cell cycle, enzyme malfunctioning, etc. [21]. 

Capecitabine (**2**) is an oral prodrug, metabolized in vivo to 5-FU by carboxylesterases, cytidine deaminase, and thymidine phosphorylase/uridine phosphorylase sequentially, which has shown effectiveness in treating advanced BC [22] and gastric cancer [23]. In early-stage TNBC patients, capecitabine in combination with standard adjuvant chemotherapy showed significant disease-free survival and overall better survival outcomes than standard chemotherapy with tolerable adverse events [24]. A study showed that the addition of capecitabine-based chemotherapy was the most effective regime [25]. 

Gemcitabine (2′,2′-difluorodeoxycytidine, dFdC) (**3**), an efficient chemotherapeutic drug for the treatment of various types of cancer in clinical practice, was also evaluated in the treatment of TNBC in several clinical trials [26]. DFdC is a potent and specific deoxycytidine analog which, once inside the malignant cell, is firstly phosphorylated by deoxycytidine kinase to its monophosphorylated form and subsequently by nucleotide kinases to its active metabolites, dFdC diphosphate (dFdCDP) and dFdC triphosphate (dFdCTP). These active metabolites are nucleosides that mediate antitumor effects. dFdCDP works as an inhibitor of ribonucleotide diphosphate reductases, an enzyme responsible for catalyzing the biosynthesis of deoxycytidine triphosphate (dCTP), which is a precursor necessary for DNA synthesis, from the corresponding ribonucleotide [27]. Therefore, the overexpression of ribonucleotide reductase is associated with the emergence of resistance to dFdC [28]. In addition, the cytotoxicity of dFdC is mainly associated with the cellular accumulation of dFdCTP. dFdCTP competes with dCTP for incorporation into DNA, thereby competitively inhibiting DNA chain elongation. This process is referred to as “masked DNA chain termination” and induces a G0/G1 and S-phase arrest in the cell cycle, which triggers apoptosis [29].

To overcome the drug resistance induced by nucleoside kinase deficiency, various therapeutic approaches have been postulated. In this context, a multisubstrate deoxyribonucleoside kinase of *Drosophila melanogaster* in the nucleus or cytosol has been reported as a potential candidate suicide gene for reversing acquired dFdC resistance in TNBC cells [30].

Standard anthracycline-based chemotherapy is the treatment of choice as first-line chemotherapy for metastatic breast cancer patients not previously treated with anthracyclines [31]. Its mechanism of action includes DNA intercalation, membrane binding, free radical formation, DNA repair cascade degradation, and cell death. Although anthracyclines represent an important component of adjuvant chemotherapy, they are associated with several short and long-term adverse events, with the major being cardiotoxicity and secondary leukemia [32,33].

Nowadays, in order to avoid unnecessary anthracycline treatment due to its associated cardiotoxicity, taxane-based regimens constitute another standard therapy for breast cancer patients, especially those affected with TNBC. Paclitaxel (PTX), isolated from the bark of the Pacific Yew, and docetaxel, a semisynthetic analog from the renewable and more readily available leaves of the European yew tree, are among the most active agents for metastatic breast cancer. The assembly promoting properties of PTX (**4**) were firstly reported in 1979 [34]. PTX, through its effects on microtubules, inhibits the growth of a variety of solid tumor cells causing, at high concentration, mitotic arrest at the G2/M phase, whereas, at low concentration, it induces cell apoptosis at the G0 and G1/S phase; however, its clinical application is limited due to poor water solubility.

Many studies have illustrated that PTX combined with 5-FU shows synergic activity and improved tolerability towards some breast carcinoma, ovarian cancer, and gastric cancer [35,36]. Chen et al. [37] have recently reported the development of KLA-modified liposomes co-loaded with 5-FU and PTX (KLA-5-FU/PTX Lps). This new drug formulation was evaluated for its antitumor activity against human breast cancer cells (MDA-MB-231). It showed enhanced cytotoxicity against MDA-MB-231 cells, improved drug delivery to mitochondria, induced mitochondria-mediated apoptosis, and turned out a promising system to target the delivery of antitumor drugs to mitochondria as a treatment for TNBC [37]. 

For patients unresponsive to the treatment mentioned above, adjuvant capecitabine or platinum-based chemotherapy, such as carboplatin and cisplatin, might be given, although it is still controversial, as researchers are currently leading an ongoing randomized phase III trial to validate the superiority of either adjuvant capecitabine or platinum-based chemotherapy [38]. 

A drug combination of gemcitabine and cisplatin (cis-diamminedichloroplatinum, cis-DDP (**5**) has proven to be superior to dFdC/PTX in first-line treatment of metastatic TNBC in terms of progression-free survival (PFS) [39]. The potential effect of nanoparticle albumin-bound (nab)-PTX and cis-DDP was assessed for metastatic mucinous adenocarcinoma [40] and metastatic TNBC [41]. Wang et al. [41] conducted a randomized phase III controlled open-label trial to compare the efficacy of nab-PTX/cis-DDP with dFdC/cis-DDP in metastatic TNBC patients. The study confirmed that the combination of nab-PTX with cis-DDP, as compared to dFdC/PTX, significantly increased the OS and led to a significantly higher objective response rate. The reduction in the risk of death may result from the higher antitumor activity of nab-paclitaxel over gemcitabine when combined with cisplatin.

Eribulin (NSC 707389) (**6**) is another medication that has been added to the armamentarium of drugs against TNBC after being studied in patients who have received at least two chemotherapeutic regimens, which include an anthracycline and a taxane. It is a non-taxane synthetic analogue of halichondrin B, isolated from the marine sponge Halichondria okadai, which acts as an irreversible inhibitor of microtubule polymerization [42]. Since it does not affect the microtubule depolymerization, it causes less toxicity compared to the previously reported taxanes [43]. This results into apoptosis through the disruption of mitotic spindles and an irreversible block of the cell cycle at the G2-M level. Currently, eribulin is used to treat HER2-negative metastatic or recurrent BC that failed previous exposure to anthracyclines and taxanes, and for treatment of TNBC as well [44]. Eribulin in combination with the PARP-1, PARP-2, and PARP-3 inhibitor olaparib showed good efficacy for advanced TNBC patients and was well tolerated [45].

## 3. Poly Adenosine Diphosphate-Ribose Polymerase

The integrity and stability of DNA in breast epithelial tissues are key factors in breast homeostasis. However, carcinogenesis is mostly initiated by DNA damage which is an ongoing process resulting from both endogenous (errors in replication) and exogenous (environmental) assaults to the human genome. Usually, cell repair mechanisms ensure that cells with damaged DNA undergo either repair or apoptosis. Therefore, inhibition of these processes can lead to a buildup of damaged DNA in cells, resulting in apoptosis or senescence of the tumor cells. Poly(ADP-ribose) polymerase (PARP) inhibitors have been closely examined as one of the most exciting and promising “targeted” therapeutic strategies to treat advanced TNBC by preventing cancer cells from repairing themselves. Indeed, it has been demonstrated that 10–20% of total patients diagnosed with TNBC have a mutation in breast cancer susceptibility genes (*BRCA*)*1* or (*BRCA*)*2* [46]. *BRCA1* and *BRCA2* are tumor suppressor genes that are associated with a hereditary predisposition to developing female breast cancer [47], but their pivotal role in the DNA damage response makes cancer cells harboring such mutations more sensitive to drugs eliciting DNA damage or interfering with DNA repair, such as PARP inhibitors.

PARPs are a family of multifunctional enzymes involved in several cellular processes, including DNA repair mechanism and apoptosis [48]. PARP-1, a nuclear, zinc-finger, DNA-binding protein that has been identified as the most abundantly expressed and characterized isoform of the PARP family, localizes to DNA strand breaks as part of the base excision repair process [49]. Upon detection of the DNA damage, PARP-1 catalyzes the addition of a poly-ADP-ribose (PAR) chain to target proteins, thus recruiting repair factors to repair DNA [50]. It is well known that PARP inhibitors are particularly effective against tumors carrying mutations in *BRCA*. In fact, it has been demonstrated that it is possible to achieve synthetic lethality and increased tumor cell death through the prevention of DNA repair via PARP inhibition, in conjunction with the loss of broken double-strand DNA repair via *BRCA*-dependent mechanisms. In other words, it has been indicated that the detection of a mutated *BRCA* gene in some TNBC patients results in the blockage of the repair process of broken single-stranded DNA via PARP inhibitors by obstructing PARP enzyme activity and PARylation reactions through the competition with coenzyme, NAD+, for the interaction with the PARP catalytic domain; in addition, *BRCA* mutants cannot induce homologues recombination to repair double-stranded DNA, with the consequence of synthetic lethal effects on tumor cells [51]. Therefore, this makes the inhibition of PARP an attractive target for TNBC tumors that are *BRCA* deficient [52]. The discovery and development of PARP inhibitors began more than 50 years ago using the nicotinamide functional group [53]. Later, PARP inhibitors were designed by introducing nicotinamide and benzamide functional groups into their structure in order to enable them to bind to the catalytic portion of PARPs [54].

### 3.1. Iniparib (***7***)

**Figure 2:**
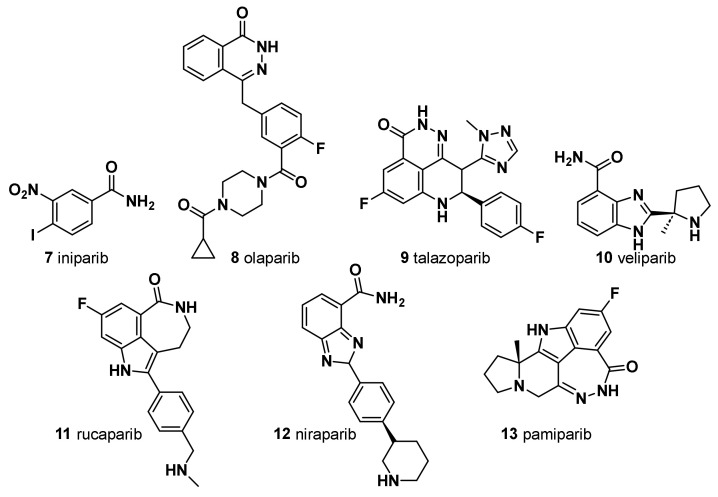
Chemical structure of PARP inhibitors.

Iniparib was evaluated as the first PARP inhibitor potential candidate agent for TNBC; however, it failed clinical trials and a definitive in vitro study revealed that the agent did not appreciably inhibit PARP [55] (Figure 2).

Here, we have reported several potential PARP inhibitors for TNBC that have been recently discovered and used in clinical progression trials.

### 3.2. Olaparib (***8***)

Olaparib was the first small-molecule PARP inhibitor to show clinical efficacy and tolerability in *BRCA1*/*BRCA2*-mutated advanced BC [56] (Figure 2). First reported as a PARP-1 and PARP-2 inhibitor, it also showed a potent PARP-3 inhibition. Olaparib and talazoparib (TALA) (**9**) were approved in 2018 by the Food and Drug Administration as monotherapy for the treatment of metastatic TNBC harboring a germline *BRCA1* (g*BRCA*) or *BRCA*2 mutation [57,58], based on the results of two OlympiADand EMBRACA clinical trials. Approximately 15% of patients with TNBC have g*BRCA* mutations, which make them good candidates as PARP inhibitors. Moreover, the use of the PARP inhibitor olaparib in the treatment of TNBC patients without *BRCA* mutations has recently been shown to be ineffective [59]. Noteworthy, in HER2-negative metastatic BC and *BRCA* mutation patients, olaparib alone has a remarkable advantage on the standard treatment. However, cancer multidrug resistance (MDR) represents a major challenge for effective cancer treatment, and overexpression of a P-glycoprotein (P-gp) and a breast cancer resistance protein (BCRP) has been hypothesized to be one of the mechanisms responsible for acquired resistance to olaparib. Thus, a second generation of PARP inhibitors has been developed and tested in several clinical trials.

### 3.3. Talazoparib (***9***)

Talazoparib shows potent PARP inhibition and trapping potential superior to other PARP inhibitors [60] (Figure 2). It achieved pathologic complete responses in germline *BRCA*-positive, HER2-negative patients with early breast cancer, including TNBC [61]. Of note, in EMBRACA clinical trials, treatment with single-agent talazoparib in patients with advanced BC and a germline *BRCA1*/*2* mutation, provided a significant PFS and a higher pathological remission rate compared to standard chemotherapy. Patient-reported outcomes were superior with talazoparib [62]. Unfortunately, even with the development of a second-generation PARP inhibitor, the rise of MDR could not be prevented. Eskiler et al. [63] have recently reported that talazoparib resistance is mediated by overexpression of BCRP and multidrug resistance-associated protein 1 (MRP1) genes in *BRCA1* mutant TNBC. In this regard, the authors, with the aim to overcome the abovementioned drug-resistance, developed novel talazoparib-solid lipid nanoparticles (SLNs), through a hot homogenization technique, as a promising therapeutic carrier to reverse MDR-mediated resistance in TNBC, showing significantly higher apoptotic rates in vitro than the free drug [63]. In fact, several in vivo and in vitro studies reported increased intracellular drug accumulation in cancer cells and effective overcoming of drug efflux-mediated resistance of SLNs.

### 3.4. Veliparib (***10***)

Veliparib is a benzimidazole, having a substitution at C-4 with a carbamoyl cluster and (2*R*)-2-methylpyrrolidin-2-yl moiety at C-2 (Figure 2). In combination with carboplatin and PTX followed by doxorubicin and cyclophosphamide, it improved the pathological complete response of patients with TNBC (but not when veliparib was added to carboplatin and PTX alone). The addition of carboplatin was considered as a potential component of neoadjuvant chemotherapy for patients with high-risk TNBC [64]. In addition, veliparib is being investigated in combination with radiation to treat patients with advanced TNBC, and thus far, the results are pending (NCT01618357).

### 3.5. Rucaparib (***11***)

A small subset of patients with high genomic loss of heterozygosity score or non-germline *BRCA1*/*2* mutation derived benefit from the PARP inhibitor rucaparib [65] (Figure 2). Currently, rucaparib is under investigation in combination with other agents such as immunotherapy, vascular epidermal growth factor receptor (VEGFR) inhibitors, or radiotherapy to treat solid tumors including TNBC (NCT03992131, NCT03542175, and NCT03911453). In 2020, a study assessing the efficacy of radiotherapy in combination with rucaparib was completed, the results of which have not yet been published.

Rucaparib camsylate (camphorsulfonate salt obtained via reaction of rucaparib with one molar equivalent of (1*S*,4*R*)-camphorsulfonic acid) has been formulated as film-coated tablets for oral route of administration. It is indicated as a monotherapy treatment of patients with advanced ovarian cancer with germline and/or somatic *BRCA* mutation who have experienced two or more chemotherapies. Rucaparib camsylate is under clinical development by Clovis Oncology and currently in phase II for TNBC [66].

### 3.6. Niraparib (***12***)

Niraparib, in a single-arm, phase II study, demonstrated promising antitumor activity and safety in patients with localized HER2-negative, *BRCA*-mutated breast cancer (Figure 2). In a pilot, single-arm, phase II study, a neoadjuvant treatment with niraparib as a single-agent demonstrated promising antitumor activity and high levels of tumor penetration in patients with HER2-negative, *BRCA*-mutated, localized BC. Niraparib showed superior tumor penetration to other PARP inhibitors [67].

### 3.7. Pamiparib (BGB-290) (***13***)

A new PARP inhibitor undergoing clinical evaluation in patients with ovarian cancer and TNBC can inhibit PARP1 and PARP2 (NCT03333915) (Figure 2). Moreover, it is being investigated for its efficacy in solid tumors including TNBC as a monotherapy and in combination with the chemotherapy agent temozolomide (NCT03150810). In a recent open-label, phase II, multicenter study in China (NCT03575065), pamiparib showed encouraging efficacy and an acceptable safety profile in patients with locally advanced and metastatic HER2 breast cancer with germline *BRCA1*/*2* mutation [68].

PARP inhibitors represent a valid therapeutical option against the TNBC (Figure 2). Novel PARP inhibitors are currently under investigation for use alone and in combination with established agents. Although *BRCA*-deficient tumors are more sensitive to PARPi due to its synthetic lethality, nearly 40% of *BRCA1/2*-deficient patients do not respond to PARPi due to the emergence of drug resistance mechanisms in TNBC. It is worth noting that the administration of PTX and doxorubicin, which are substrates of the MDR1 transporter, prior to PARPi treatment may lead to the upregulation of MDR1 and indirectly induce PARP resistance. In a specific study, the use of paclitaxel before PARPi was found to be significantly linked to the presence of ABCB1 fusion transcripts [69]. However, the use of doxorubicin before PARPi did not show a significant association with ABCB1 fusion transcripts. Other studies have also demonstrated the existence of cross-resistance between MDR1, PARPi, and paclitaxel [70,71]. Therefore, conversely, the occurrence of MDR1 overexpression as a mechanism of resistance to PARPi has implications for the choice of subsequent treatment following PARPi resistance. 

An even greater challenge is to decipher the mechanisms of intrinsic PARP inhibitor resistance among patients with g*BRCA*m. Therefore, there is still a need to improve the therapeutic options for TNBC patients, especially in specific TN molecular subtypes [72].

## 4. Cyclin-Dependent Kinases

Cyclin-dependent kinases (CDKs) are an evolutionary conserved serine/threonine protein kinases family which do not possess autonomous enzymatic activity but need to be bound to a cyclin partner to function properly. The human genome encodes 20 CDKs, divided into two subfamilies: cell cycle-associated CDKs (CDK1−7 and CDK14−18) and transcription-associated CDKs (CDK7−13, 19, and 20). CDKs are master regulators of cell cycle control, transcription, and pre-mRNA processing, and their proper activity ensures genetic integrity, cell division, and cell cycle regulation [73,74,75]. Hence, it appears clear their relevance in tumor cell proliferation and growth, which has prompted, for more than 25 years so far, the development of small molecules inhibiting their oncogenic activity.

### 4.1. CDK4 and CDK6 Inhibitors

Within the CDKs family, inhibitors of cyclin-dependent kinase 4 (CDK4) and 6 (CDK6) have been long considered as the most relevant drug candidates for cancer therapy due to their potential to restore control of the cell cycle [76]. CDK4 and CDK6 regulate progression through the cell cycle G1 phase and cell cycle initiation. In detail, both kinases form a complex with cyclin D1 that phosphorylates the tumor suppressor protein retinoblastoma (Rb) (or its homologs, p107 and p130). This activity blocks the growth inhibitory function of Rb; indeed, phospho-Rb (pRb) releases its grip and activates the transcription factors E2F, allowing the transcription of genes which are crucial for cell cycle progression to the S phase [77]. Overexpression of cyclin D1 characterizes almost 50% of BC tumors [78], and nearly 30% of TNBC presents Rb dysfunction [79]. According to The Cancer Genome Atlas, cyclin D1 amplification is preferential to luminal-type tumors, especially luminal B [80].

**Figure 3:**
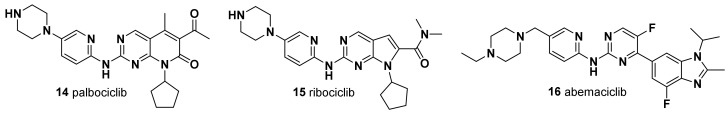
Chemical structure of CDK4 and CDK6 inhibitors.

The first generation of CDK inhibitors (CDKi) (e.g., flavopiridol, olomucine, and roscovitine), which were designed to halt cell cycle and cell proliferation by blocking CDK enzymatic activity, suffered from poor selectivity and high toxicity in normal cells. Successively, a second generation of CDKi (e.g., dinaciclib an CYC065) has been developed with greater selectivity and fewer side effects [81]. Finally, among the third generation of CDKi, in March 2017, selective CDK4/6 inhibitors received FDA approval for the treatment of post-menopausal women with hormone receptor (HR)-positive metastatic breast cancer, in combination with an aromatase inhibitor as initial endocrine-based therapy. Currently, the three FDA- and EMA-approved CDK4/6 inhibitors are palbociclib (**14**), ribociclib (**15**), and abemaciclib (**16**) (Figure 3).

#### 4.1.1. Palbociclib (PD-0332991, **14**)

Palbociclib is a highly selective cyclin-dependent kinases 4 and 6 (CDK4/6) inhibitor which hampers the phosphorylation of CDK4/6, resulting in hypophosphorylation of Rb and in the blockage of cell cycle progression from the G1 to the S phase, thus preventing DNA synthesis required for cellular replication [82]. Palbociclib has been shown to inhibit the growth of a panel of TNBC cell lines, albeit with a lesser extent than ER-positive cell lines [82]. 

Although CDK 4/6 inhibitors are a feasible option for BC therapy, resistance to these inhibitors represents a significant barrier to their effective use. It has been reported that non-luminal/basal subtypes were the most resistant to palbociclib [83]. One of the potential drug resistance mechanisms observed in 7–20% of TNBC patients is the decline in Rb1 expression after being exposed to CDK4/6 inhibitors [84]. Therefore, considering the frequent occurrence of Rb downregulation in TNBC, which results in the accumulation of p16ink4a, the use of CDK4/6 inhibitors alone may not be the optimal choice of treatment for TNBC patients [85]. To date, the most significant successes of overcoming resistance in patients with metastatic ER+/HER2 cancer, are represented by the combination of CDK4/6 inhibitors with endocrine therapies [86,87]. 

A screening on multiple TNBC cell lines, representative of the heterogeneity of this subtype, revealed the luminal androgen receptor subgroup as the most sensitive to palbociclib-mediated CDK4/6 inhibition [84]. In this regard, Tseng et al. found that palbociclib effectively inhibited retinoblastoma-proficient TNBC cell growth, and the expression of androgen receptors might contribute to palbociclib-mediated G1 arrest [88]. Moreover, they showed that treatment with the androgen receptor antagonist enzalutamide enhanced the palbociclib-induced cytostatic effect in androgen receptor-positive/retinoblastoma-proficient TNBC cells. 

#### 4.1.2. Ribociclib (LEE011, **15**)

Similar observations have been made for ribociclib, another orally accessible, selective, small-molecule inhibitor of CDK4/6 that prevents cell cycle progression and induces G1-phase arrest by inhibiting the phosphorylation of the retinoblastoma protein. Indeed, Hosseini et al. recently evaluated the long-term consequences of the treatment of ribociclib in combination with enzalutamide [89]. They demonstrated that the combination of ribociclib with enzalutamide in an androgen receptor-positive model (MDA-MB-231 and MCF-7 cell lines) results in a more effective improvement in the cell cytotoxicity than the separate treatments, suggesting a potential approach for enhancing antitumor effectiveness. Collectively, these observations suggest that combined inhibition of CDK4/6 and AR is a promising therapeutic strategy for androgen receptor-positive/retinoblastoma-proficient TNBCs.

The activation of alternative proliferative pathways, such as mTOR and PI3K, and the deregulation of D-type cyclins expression represent further mechanisms of resistance to CDK4/6 inhibitors [87,90]. More recently, sequestration of CDK4/6 inhibitors into tumor cell lysosomes was shown to be another evasion mechanism more active in TNBC cells than in other BC cell lines, which might be counteracted by the concomitant administration of lysosome-destabilizing compounds [91]. Remarkably, inhibition of a CDK, cyclin-dependent kinase 2 (CDK2), which, in association with cyclin E, regulates the transition from the G1 to the S phase, synergically with CDK4/6, was shown to sensitize resistant TNBC cells to CDK4/6 inhibition [87,91].

#### 4.1.3. Abemaciclib (**16**)

As well as all of the new generation selective CDK4/6 inhibitors covered in this review, abemaciclib, an approved monotherapy, also plays an important role in G1–S phase transition in the cell cycle. Although preclinical and clinical studies have shown the therapeutic potential of abemaciclib in pretreated HR^+^, HER2^−^ breast cancer patients, compared to other CDK4/6 inhibitors, its potential effects on TNBC therapy have not been definitively elucidated. Recently, Sekeroglu et al. demonstrated that abemaciclib caused significant apoptosis in TNBC cells via G0/G1 arrest, chromatin condensation, the upregulation of caspase-3 and Bax levels, and the downregulation of Bcl-2. The formation of a large number of cytoplasmic vacuoles not associated with autophagy suggested that treatment with **16** could be effective for TNBC [92] (Figure 3).

In a study published in 2019 by Petronini et al., it has been also highlighted that the sequential treatment of palbociclib for 24 h followed by PTX for a further 48 h in both MDA-MB-231 and HCC38 TNBC cell lines produced an additive inhibitory effect on cell proliferation associated with a significant increase in apoptotic cell death [93]. The efficacy of this schedule relies on the reversible action of palbociclib on the cell cycle; in fact, upon palbociclib removal, the cells arrested in the G1 phase synchronously re-enter the cell cycle in the S phase, becoming more sensitive to the cytotoxic effect of PTX. On the other hand, it has been demonstrated that the simultaneous combination of palbociclib with increasing concentrations of PTX give rise to an antagonistic effect, highlighting the importance of the successful use of sequential therapy in order to minimize the insurgence of adverse effects and to allow patients to derive benefit from the additional treatment. [93,94]. Indeed, the above-mentioned antagonism could be ascribed to the reduced activity of cytotoxic chemotherapeutic agents, directed against cycling cells, when used in cells already arrested in the G0/G1 phase.

Moreover, the pretreatment of TNBC cells with palbociclib led to an increase in sensitivity to cisplatin therapy, enhancing the anticancer activity. Therefore, these results suggest that the sequential use of CDK4/6 inhibitors with SOC agents might provide an alternative therapeutic approach for TNBC [95].

### 4.2. CDK2 Inhibitors

#### 4.2.1. Roscovitine (**17**)

**Figure 4:**
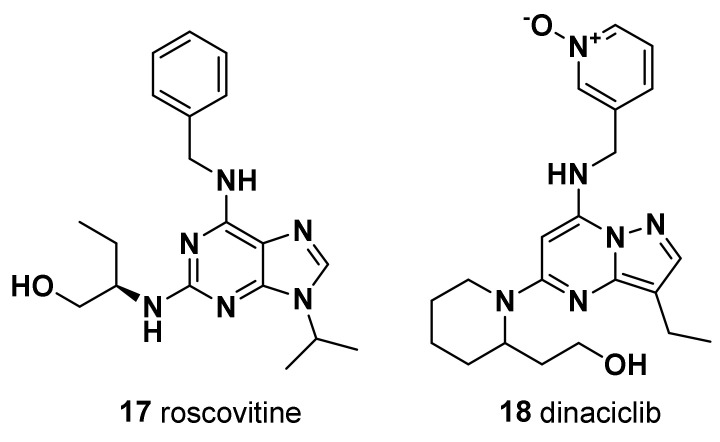
Chemical structure of CDK2 inhibitors.

Similarly, sensitizing effects to taxanes in TNBC cells were shown for the CDK2 inhibitor roscovitine [96], which was also shown to interfere with TNBC cell migratory and metastatic properties [97] (Figure 4). 

#### 4.2.2. Dinaciclib (**18**)

In addition, CDK2 inhibition via dinaciclib treatment was shown to promote reactivation in TNBC cells of the ER expression, rendering them sensitive to antiestrogen therapy, such as tamoxifen [98] (Figure 4). This latter activity was independent of the prominent role of CDK2 in cell cycle regulation but was correlated to its ability to phosphorylate the epigenetic transcriptional regulator EZH2 [98]. However, clinical trial probing safety and efficacy of the combination of dinaciclib with anthracyclines failed due to its high toxicity and poor response [99]. Thus, the development of novel specific CDK2 inhibitors represents an open field of study, with high potential for TNBC pharmacotherapy. 

### 4.3. CDK7 and CDK12 Inhibitors

Among the transcriptional CDKs ensuring proper progression of the transcription cycle and its accurate coordination with the pre-mRNA processing events, CDK7 is one of the most promising therapeutic targets for TNBC treatment. CDK7 associates with cyclin H and is part of the general transcription factor TFIIH, which regulates transcription initiation by phosphorylating both the C-terminal domain (CTD) of the RNA polymerase II (RNPII) at Ser5 and CDK9, the catalytic subunit of the transcription elongation factor P-TEFb [75]. TNBC was shown to be highly sensitive to chemical inhibition of CDK7 activity via treatment with the inhibitors THZ1 and its derivative THZ2, which significantly display growth inhibitory effects in both TNBC cell lines and xenograft models [100]. Sensitivity to these inhibitors was correlated to a transcriptional addiction of TNBC to CDK7, whose activity ensures proper expression of genes essential for TNBC tumorigenicity [100], including condensin genes guaranteeing chromosome stability [101]. Coherent with CDK7’s essential role for TNBC cell survival, high expression levels of this kinase are a marker of poor prognosis for TNBC patients [102].

#### 4.3.1. Samuraciclib (ICEC0942; CT7001) (**19**)

**Figure 5:**
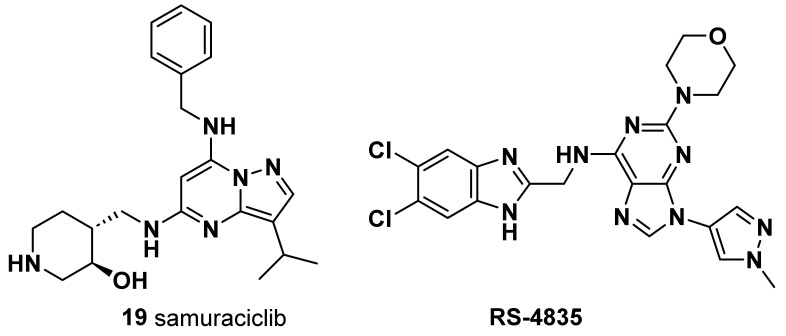
Chemical structure of CDK7, CDK12, and CDK13 inhibitors.

A small-molecule, adenosine triphosphate (ATP)-competitive inhibitor of CDK7, orally available, showed tolerable safety and partial clinical benefit for TNBC patients, thus supporting further studies evaluating its potential as a novel therapeutic strategy [103] (Figure 5).

Although among the other transcriptional kinases only CDK9 was found to be another essential gene for TNBC cell survival [100], CDK12 also represents a therapeutic target of high interest for this tumor subtype. CDK12, together with its highly homologous CDK13 associate with cyclin K, regulates transcriptional elongation by mediating RNPII CTD phosphorylation at Ser2 in the gene body and towards the 3′-end of the transcription units [75].

#### 4.3.2. SR-4835

CDK12 and CDK13 were found to be essential for the proper splicing and the prevention of premature intronic polyadenylation of pre-mRNAs encoded by long genes, thus ensuring their proper expression [104,105] (Figure 5). Notably, expression of DNA damage response (DDR) genes was shown by multiple studies to be highly sensitive to CDK12/13 depletion and/or inhibition [104,105,106,107]. Impaired pre-mRNA processing and consequent downregulation of the expression of genes in the DDR pathway caused by treatment with SR-4835, a selective dual inhibitor of CDK12 and CDK13, was shown to elicit in DNA repair-proficient TNBC cells, such as *BRCA1*/*2* wild-type cells, a “BRCAness” condition which increases their sensitivity to DNA-damaging chemotherapy and PARP inhibitors [108]. By eliciting similar downregulation of DDR genes, profound antiproliferative effects and synergy with PARP inhibitors in TNBC cells were also shown via treatment with PROTAC degraders targeting either both CDK12 and CDK13 [109] or specifically CDK12 (PP-C8) [110]. CDK12/13 inhibitors represent an important innovation in the field of TNBC chemotherapy, as they might allow administration of PARP inhibitors also to tumors displaying either innate (i.e., *BRCA1*/*2* wt) or acquired resistance to these molecules [107,108]. In this regard, particular interest is merited by some recently developed compounds able to simultaneously inhibit CDK12 and PARP1 activity, whose antitumoral activity in TNBC cells has just been preliminarily tested [111]. Nevertheless, as the broad transcriptional and post-transcriptional changes are elicited by CDK12/13 inhibition in TNBC, it is conceivable that such treatment might elicit other actionable vulnerabilities in these tumors, as recently shown by analogous treatment in high-grade serous ovarian cancer [112]. 

Overall, the pivotal role that CDKs exert in controlling the mammalian cell cycle and transcription and their checkpoints raises the possibility of devising therapeutic strategies based on the druggability of these molecules.

## 5. Microtubules

In addition to the development of PARP and CDK inhibitors, another strategy for the treatment of patients with TNBC involves the employment of microtubule-targeting agents. Microtubules play critical roles in a wide number of cellular functions, such as motility, division, shape maintenance, and intracellular transport. The major protein component found in microtubules is tubulin. Microtubule-targeting agents (MTAs) inhibit the function of cellular microtubules by promoting polymerization and depolymerization, resulting in the arrest of cell cycle progression and induction of cell death [113,114].

Existing tubulin inhibitors, such as PTX, have shown great effectiveness in treating breast cancer; however, their use is limited by the need for intravenous administration due to poor aqueous solubility. In addition, their clinical use is often limited by drug resistance mediated by ABC transporters and neurotoxic side effects. Extensive preclinical study investigations have suggested that tubulin inhibitors designed to target the colchicine binding site are notably less susceptible to transporter-mediated drug resistance [115]. Although, up to this point, none of these colchicine binding site inhibitors (CBSIs) have gained FDA approval, mainly due their toxicity profiles and limited clinical efficacy; several novel CBSIs have been developed as alternatives to PTX and tested in clinical trials. 

### 5.1. VERU-111

**Figure 6:**
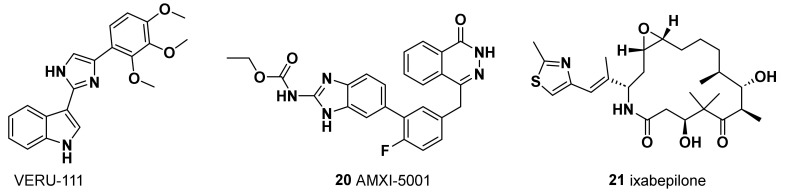
Chemical structure of microtubule inhibitors.

A series of compounds, termed ABI-III chemotypes, were first reported in 2012 by Chen et al. as potent tubulin polymerization inhibitors able to overcome ABC transporter-mediated multidrug resistance [116]. More recently, Deng et al. [117,118] and Kutrilina et al. [119] evaluated in TNBC models the preclinical safety and efficacy of a novel, potent, and orally bioavailable tubulin inhibitor, VERU-111 (also known as sabizabulin, Figure 6) which is currently undergoing clinical trials. The authors reported strong antiproliferative activity in MDA-MB-231 and MDA-MB-468 TNBC cell lines, with an IC_50_ value in the low nanomolar range, overcoming P-gp-mediated multidrug resistance, which frequently develops in patients treated with conventional taxanes [118,119]. In addition, in vivo assays in two aggressive TNBC xenograft models demonstrated its potent anticancer activity, its antimetastatic potential, and the reduction in adverse side effects relative to PTX [118,119]. Therefore, based on these findings, VERU-111 represents a promising alternative agent to target tubulin in patients with advanced breast cancer, with similar antimetastatic efficacy to PTX but with the advantage of oral bioavailability and lower toxicity than taxanes. 

On the other hand, a synergistic role of PARP and microtubule-targeting agents is also emerging in cancer therapy. Simultaneous targeting of PARP and microtubule polymerization may result in amplified and prolonged DNA damage, an enhanced sensitization of cancers, and a broad antitumor efficacy, with reduced risk for both cancer drug resistance and dose-limiting peripheral neuropathy associated with microtubule-targeting agents [120,121]. In this context, many efforts have been made to design and synthesize, through a medicinal chemistry approach, novel bifunctional small molecules able to synchronously inhibit the catalytic activity of PARP1 and microtubule polymerization.

### 5.2. AMXI-5001 (***20***)

AMXI-5001 was therefore engineered as a dual microtubule polymerization and PARP1/2 inhibitor, with favorable metabolic stability, oral bioavailability, and pharmacokinetic properties (Figure 6). AMXI-5001 showed inhibition of both tubulin polymerization, comparable to the vinblastine, and PARP inhibition at the level of PARP inhibitors **7**–**13**. AMXI-5001 showed antitumor activity in a BRCA-mutated TNBC model in vivo as well, achieving complete tumor regression upon oral administration. Compared to the current PARP1 and 2 inhibitors, AMXI-5001 induced antitumor cell responses and elicited DNA repair biomarkers at much lower concentrations. The antitumor effect of AMXI-5001 was superior to PARP or microtubule inhibitors, both alone and a combination of both agents [120].

### 5.3. Ixabepilone (BMS-247550) (***21***)

Although there is no apparent mechanistic basis for unique sensitivity of microtubule-stabilizing agents in this subgroup, ixabepilone may have a role in the treatment of TNBC. Ixabepilone is a semisynthetic epothilone B analogue that behaves as microtubule stabilizer (Figure 6). Epothilones are cytotoxic macrolides with a mechanism of action similar to PTX but with the potential advantage of activity in taxane-resistant settings in preclinical models. The antineoplastic effect of epothilones has been ascribed to the stabilization of microtubules, which results in mitotic arrest at the G2/M transition. The effect of ixabepilone in combination with capecitabine in patients with metastatic or locally advanced TNBC was analyzed in two phase III trials (NCT00080301 and NCT00082433). The results showed that the drug combination, compared with capecitabine alone, improved PFS and the objective response rate in patients with advanced TNBC previously treated with anthracyclines and taxanes [122].

## 6. Mitotic Kinase Inhibitors

Small molecules inhibiting the activity of mitotic kinases have been long studied as alternative antimitotic agents to microtubule-targeting drugs, which still present remarkable toxicity issues. Mitotic kinase inhibitors retain high potential as novel therapies for TNBC, as several of these kinases [i.e., Aurora kinase A (AURKA) and B (AURKB), Polo-like kinase 1 (PLK1), NIMA-related kinase 2 (NEK2), and mitotic checkpoint kinase Mps1/TTK] have been shown to be highly expressed in these tumors, compared to both normal tissue and other BC, and to be prognostic for chemoresistance and worse outcomes [123,124,125,126,127,128].

AURKA is the mitotic kinase that records the largest number of preclinical and clinical studies testing the antitumoral activity of its select inhibitors in TNBC. AURKA is a serine–threonine kinase associated to the centrosome and the spindle microtubules, whose activity is pivotal to the proper progression of mitosis.

### 6.1. Alisertib

**Figure 7:**
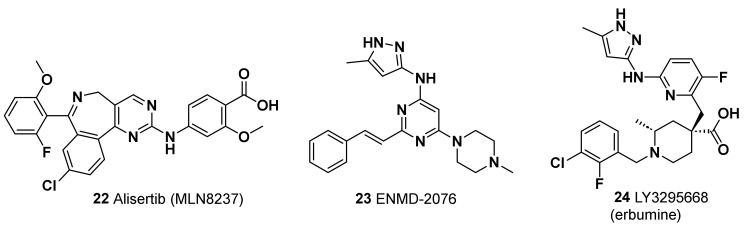
Chemical structure of AURKA inhibitors.

One of the first selective inhibitors of AURKA tested in TNBC was Alisertib (MLN8237, **22**) (Figure 7), an orally available molecule showing a potent ad selective activity against its target (IC_50_ = 1 nM) and 200-fold more selectivity with respect to the close-related AURKB [129]. Preclinical studies showed that the combination of alisertib with either conventional taxanes [130] or with the new generation microtubule inhibitor eribulin (**5**) [131] reduces the growth and metastatic spreading of TNBC xenograft models. Coherent with these results, different clinical studies showed a significant improvement in PFS in advanced and metastatic BC treated with alisertib in addition to PTX [132,133]. Increased PFS and OS were observed for this therapeutic combination also for TNBC patients, although the cohort tested did not reach an adequate size for a powered statistical analysis [132]. These observations raise hopes that alisertib could be added to currently available therapeutic regimens for TNBC in order to improve their efficacy. In addition to its canonical mitotic role, AURKA was also shown to positively regulate activation of the protumoral mTOR pathway in TNBC cells. This uncanonical function of AURKA underlies the enhancing effects shown by two distinct mTOR inhibitors, rapamycin and TAK-288, on the antitumoral activity of alisertib against TNBC xenograft models [134,135]. Notably, alisertib combination with TAK-288 was proven to be tolerable in patients with advanced solid tumors in a phase I clinical trial [134]. Since other mitosis-unrelated functions, such as regulation of gene expression and RNA splicing, have been described in different cancers for AURKA, as well as for another centrosomal kinase NEK2, [126,136,137,138], further characterization of their activity and of the molecular profile of patients displaying their altered expression could improve the rationale design of novel therapeutic strategies exploiting their inhibitors.

### 6.2. ENMD-2076

Another AURKA inhibitor with proven therapeutic efficacy against TNBC, both in preclinical and clinical studies, is the multitarget molecule ENMD-2076 (**23**, Figure 7) [139,140]. This orally available drug is a multitarget inhibitor, more effective for AURKA IC_50_ = 14 nM) with respect to AURKB (IC_50_ = 350 nM), that also targets the receptor-type tyrosine-protein kinase FLT3 (IC_50_ = 3 nM). A phase II clinical trial enrolling patients with locally advanced or metastatic TNBC previously treated showed 6 months of clinical benefits in 16.7% of patients treated with ENMD-2076 as single agent, with partial response and stable disease. These results encourage further studies investigating the potential therapeutic inhibitors for TNBC patients in combination with other agents. Furthermore, as TP53 gene mutations and overexpression were found to enhance TNBC cell line sensitivity to ENMD-2076 in a preclinical study [140], it is conceivable that the therapeutic efficacy of this drug could be improved via administration to molecularly stratified patients.

### 6.3. LY3295668

The AURKA inhibitor most recently tested against TNBC tumor is LY3295668 (erbumine, **24**, Figure 7), an orally available inhibitor of AURKA, with over 1,000-fold selectivity versus AURKB (K_i_ of 0.8 nM and 1038 nM for AURKA and AURKB, respectively). This inhibitor was developed following the results of a pharmacogenomics screening identifying synthetic lethality between AURKA inhibition and RB1 mutations in a wide panel of cancer cell lines [141]. Remarkably, LY3295668 displayed a high antitumoral activity and a low toxicity in murine xenograft models of small-cell lung cancer [141]. In addition, a phase 1 study in patients with locally advanced or metastatic solid tumors showed that LY3295668 has a manageable toxicity profile, with 69% of the patients showing a stable disease (NCT03092934, [142]). LY3295668 (**24**) thus represents a highly promising therapeutic agent for the treatment of TNBC patients who, as mentioned above, display a high rate (~30%) of RB1 dysfunction. Remarkably, a loss of RB1 was demonstrated to also sensitize TNBC cells to the antitumoral activity of several inhibitors for another centrosomal kinase, PLK1 (i.e., BI-2536, HMN-214, and Volasertib [143]). These observations strongly indicate that mitotic kinase inhibitors are a relevant alternative therapeutic strategy for TNBC and other BC which, because of their genetic status, might display intrinsic or acquired resistance to CDK4/6 inhibitors. 

Several inhibitors targeting the activity of mitotic kinases displaying aberrant expression and oncogenic activity in TNBC cells have been tested in preclinical studies and showed promising efficacy in interfering with the proliferative, invasive, and chemoresistant phenotype of these cells (Table 1). However, few of these drugs have been clinically tested in TNBC, making their further development and investigation an open field for the unveiling of novel routes of therapeutic intervention against TNBC.

## 7. Antibody Drug Conjugates

Antibody–drug conjugates (ACDs) are immunoconjugate agents engineered to selectively deliver small molecules to a cancer cell. This novel approach combines the specificity of a monoclonal antibody (mAb) with the high potency of small molecules with a lower rate of the undesirable side effects of antineoplastics.

In order to design ACDs from a medicinal chemistry point of view, it is necessary to combine three components in the chemical structure: a monoclonal antibody directed to a selective tumor antigen; a highly potent antineoplastic agent; a linker that binds the former two entities. The resulting drug is administrated intravenously to avoid the degradation via gastric acid, and after binding the target antigen expressed on the surface of cancer cells, it subsequently forms a drug–antigen complex which undergoes internalization with the cell via receptor-mediated endocytosis. Hence, an early endosome is firstly formed, and it successively fuses with the cell lysosome, which contains proteases and undergoes lysosomal degradation. The cleavage of the linker due to acidic pH, as a result of the influx of proton ions into the endosome or due to the presence of protease in the lysosome, allows the release of payloads into the cytoplasm and the payloads to take effect. 

Recent studies have examined ACDs to potentially replace a chemotherapy backbone as the preferred therapeutic partner for treatment of TNBC [170].

### 7.1. Mirvetuximab Soravtansine (***25***)

**Figure 8:**
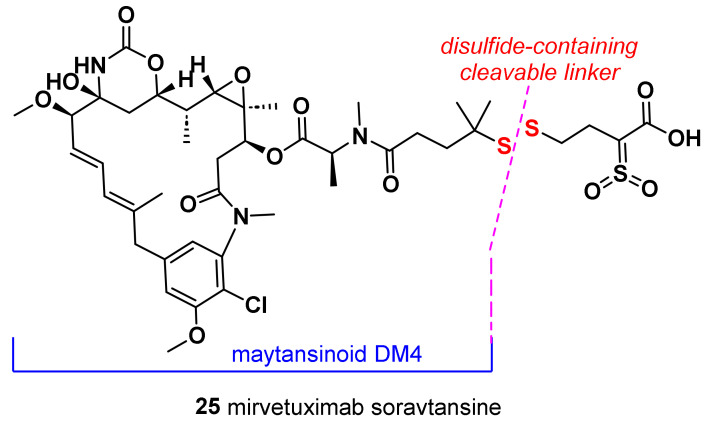
Chemical structure of mirvetuximab soravtansine.

Mirvetuximab soravtansine is an immunoconjugate composed of the humanized monoclonal antibody M9346A, targeting the folate receptor α (FRα) and conjugated to the cytotoxic maytansinoid DM4 via the disulfide-containing cleavable linker (Figure 8). Maytansinoid DM4 is a cytotoxic molecule which binds to tubulin through a second metabolite called *S*-methyl-DM4 and inhibits tubulin polymerization and microtubule assembly, resulting in cell cycle arrest and death. 

The anti-FRα monoclonal antibody moiety of mirvetuximab soravtansine targets and binds to the cell surface antigen FRα. FRα has been reported to be expressed in up to 80% of TNBC, with limited expression in normal tissues. Mirvetuximab soravtansine was evaluated in a lead-in cohort to establish its activity in patients with metastatic TNBC [171]. A phase II study of **25** was conducted in TNBC; however, this study was terminated early due to the low rate of FRα positivity in the screened patient population and the lack of disease response in the two patients treated [172].

### 7.2. Praluzatamab Ravtansine (***26***)

**Figure 9:**
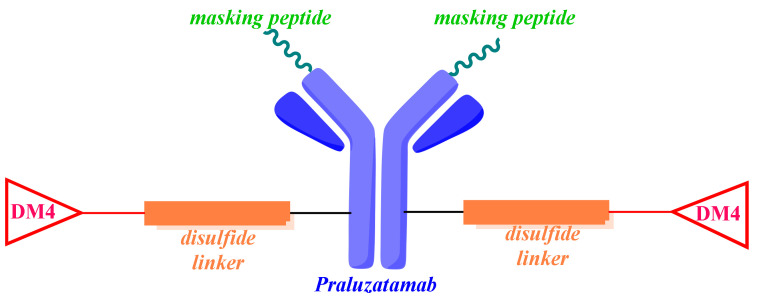
Chemical structure of praluzatamab ravtansine.

Praluzatamab ravtansine, also known as CX-2009, is a conditionally activated probody–drug conjugate that demonstrates both translational and clinical activity in a variety of tumor types [173]. A probody therapeutic candidate consists of three components: a monoclonal antibody directed against a specific tumor antigen; a peptide able to mask the complementarity-determining regions; a protease-cleavable substrate that acts as a linker between the former components. Therefore, CX-2009 is composed of praluzatamab, a monoclonal antibody targeting the activated leukocyte cell adhesion molecule (ALCAM/CD116), conjugated to the potent microtubule inhibitor DM4 via a disulfide cleavable linker (Figure 9) [174]. The activated leukocyte cell adhesion molecule (ALCAM), known as CD166, is an attractive target for the activated drug conjugate [175]. CD166 is overexpressed at the outer cell surface of multiple tumor types (breast, prostate, lung, and ovarian cancers) and, to a lesser extent, by healthy tissue (colon, stomach, pancreas, thyroid, uterus, and prostate) [176]. With the aim of avoiding the binding of the monoclonal antibody to the CD116 antigen expressed in healthy cells, the binding area of this antibody is masked by a cleavable peptide (Figure 9). Only the proteases localized in the tumor microenvironment can cleave the linker and cause the release of the complementarity-determining region-masking peptide. Then, the monoclonal antibody binds to the CD116 antigen on the surface of tumoral cells, the ADC is then internalized, and the DM4 payload is released intracellularly. 

In preclinical studies, CX-2009 demonstrated selective tumor targeting, with reduced uptake in normal tissue. Moreover, it has been reported in the phase 2 CTMX-2009-002 trial (NCT04596150) that CX-2009 generated responses in HR-positive, HER2-negative breast cancer; however, it did not meet the primary end point in patients with TNBC [177].

### 7.3. SAR566658

*SAR566658* is another humanized DS6 (huDS6) antibody directed against tumor-associated sialoglycotope CA6 (tumor-associated glycosylated MUC-1) and conjugated through a cleavable linker to the cytotoxic maytansinoid-derived drug DM4 [178]. It binds to tumor cells with high affinity, allowing good intracellular delivery of DM4. The CA6 sialoglycotope of MUC1-glycoprotein is highly detected on various tumors of epithelial origin (pancreas, ovary, breast, and bladder) [179]. SAR566658 showed in vivo antitumor efficacy against CA6-positive human pancreas, cervix, bladder, and ovary tumor xenografts and against three breast patient-derived xenografts. A phase I dose expansion was conducted to evaluate the safety and the maximum tolerate dose of SAR566658. A phase II study was then conducted in patients with CA6-positive mTNBC. Preliminary results have shown an unfavorable benefit/risk balance due to a higher-than-expected incidence of ophthalmologic events (e.g., keratitis and keratopathy) [178].

### 7.4. Sacituzumab Govitecan

Sacituzumab govitecan, also known as IMMU-132 and marketed as Trodelvy^®^, was approved by the FDA in April 2020 for the treatment of metastatic triple-negative breast cancer that did not respond to at least two prior therapies for metastatic disease [180]. It is an ADC drug composed of SN-38, the active metabolite of irinotecan (CPT-11) with a cytotoxic effect by inhibiting DNA topoisomerase I, that is conjugated via a pH cleavable linker to a humanized IgG1 antibody targeting trophoblastic cell-surface antigen 2 (TROP-2), a transmembrane glycoprotein that participates in the proliferation, survival, and invasion of tumoral cells and whose overexpression has been related to a poor prognosis. Sacituzumab govitecan-bound tumor cells are killed via intracellular SN-38 uptake, while nearby tumor cells are killed by extracellular SN-38 release. In a recent study, the efficacy and safety profile of IMMU-132 as a second-line treatment for refractory mTNBC have been highlighted; however, its utility is limited due to inadequate intratumor exposure and dose-limiting toxicities, as is the case with other topoisomerase-1 inhibitors [181].

## 8. PI3K

The phosphatidylinositol-3-kinases (PI3Ks) are intracellular signaling enzymes that phosphorylate the free 3-hydroxyl of the phosphoinositides in the cell membrane. Therefore, this signaling pathway is involved in key cellular functions, such as cell proliferation, migration, survival, and metabolism [182]. PI3Ks are formed of a heterodimer composed by a regulatory (p85) and a catalytic (p110) subunit and are usually grouped in different classes, among which class I is the most commonly altered in cancer. In cancer cells, activation of the PI3K pathway is caused by molecular alterations, including gene mutations encoding the PI3K alpha (*PIK3CA*) and beta (*PIK3CB*) catalytic subunits, AKT1 mutations, and a loss of expression of phosphatidylinositol-3,4,5 trisphosphate (PIP3), phosphatase and tensin homologue (*PTEN*), and inositol polyphosphate-4-phosphatase type II B (INPP4B). *PIK3CA* is the second most frequently mutated gene after *TP53* in TNBC, with additional inactivating alterations in *PTEN* and additional activating mutation in *AKT1* [80].

PI3K inhibitors are divided into pan-PI3K inhibitors (buparlisib, pictilisib, and copanlisib) and isoform-specific PI3K inhibitors (alpelisib). Pan-PI3K inhibitors target all four isoforms (alpha, beta, gamma, and delta) of the class-IA PI3K p110 catalytic subunit.

### 8.1. Buparlisib (BKM120, ***27***)

**Figure 10:**
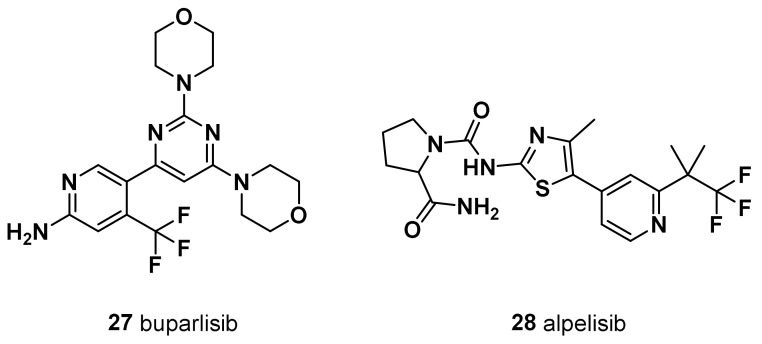
Chemical structure of PI3K inhibitors.

Buparlisib is one of the furthest developed pan-class I PI3K inhibitors in combination with chemotherapeutic agents (Figure 10). It is orally bioavailable and acts to inhibit PI3K in an ATP-competitive manner [183]. In the phase 1 study, buparlisib was administered at max dose of 100 mg/die and yielded partial response in a TNBC patient [184,185]. In a phase 2 clinical study, buparlisib monotherapy achieved prolonged stable disease in a small subset of TNBC patients (no strong clinical signal of efficacy was observed). Actually, buparlisib is under clinical development by Adlai Nortye Biopharma and more specifically in phase II for TNBC. According to GlobalData, phase II drugs for TNBC have a 25% phase transition success rate indication benchmark for progressing into phase III [186]. It has also been observed that, compared to selective p119-alpha inhibitors, buparlisib shows a toxicity profile similar to other pan-PI3K inhibitors due to its elevated CNS penetration or to other pharmacokinetic differences [183]. Furthermore, dual PI3K and PARP inhibition with buparlisib and olaparib (**8**) reduced the tumor growth in patient-derived primary tumor xenografts, displaying *BRCA1*/*2* downregulation following PI3K inhibition in TNBC [187].

### 8.2. Alpelisib (***28***)

Alpelisib is an α-specific PI3K inhibitor that has been evaluated as a monotherapy regimen in patients with advanced PI3K pathway mutant ER+ HER2-negative BC and TNBC (Figure 10). Alpesilib displayed efficacy in heavily pretreated ER+ breast cancer with *PIK3CA* mutation [188]. Alpelisib in combination with olaparib (**8**) is tolerable in patients with pretreated TNBC, with evidence of activity in non-*BRCA* carriers. The results highlight the potential synergistic use of a PI3K inhibitor to sensitize HR-proficient (BRCA wild-type) TNBC to a PARP inhibitor [189]. A phase II trial study has been established to evaluate the drug combination of alpelisib and nab-paclitaxel in treating patients with TNBC with *PIK3CA* or *PTEN* alterations who do not respond to anthracycline chemotherapy (anthrocycline refractory) [190].

In addition, it is worth mentioning that nearly 35% of TNBCs have become resistant to PI3K/AKT/mTOR inhibitors due to the loss of *PTEN* gene expression [191], which encodes a phosphatase protein that returns the PI3K/Akt/mTOR pathway to its inactivated state [192]. The lack of *PTEN* gene expression has been correlated to the emergence of the glycolytic phenotype, known as the Warburg effect [193]. However, it has been demonstrated that drug combinations may effectively contrast the multidrug resistance [194]; in fact, a positive therapeutic response of *PTEN*-deficient TNBC was observed by combining PI3K and histone demethylase lysine demethylase 4B (KDM4B) inhibitors and suppression of either the 3-phosphoinositide-dependent kinase 1 or se-rum/glucocorticoid-regulated kinase 1 activity [195]. The drug combination of chemo-therapeutic agents or neoadjuvant chemotherapy with the monoclonal antibody bevacizumab was shown to improve pathological complete response in stage II to III TNBC [196,197].

## 9. AKT

The serine/threonine kinase AKT, also known as protein kinase B (PKB), is a key component of the phosphatidyl-inositole-3 kinase (PI3K) intracellular pathway that exerts a pivotal role in cell growth, survival, proliferation, and metabolism [198,199,200,201]. The PI3K/AKT signaling pathway has been found to be hyperactivated in BC and is considered a promising target for TNBC [202]. Three isoforms of AKT have been identified so far: AKT1 (also known as PKBα), AKT2 (also known as PKBβ), and AKT3 (also known as PKBγ). In cancer cells, AKT1 is involved in proliferation and growth, promoting tumor initiation and suppressing apoptosis; AKT2 regulates cytoskeleton dynamics, favoring invasiveness and metastatization, whereas the role of the hyperactivation of the AKT3 isoform in cancer is still controversial, although a possible stimulation of cell proliferation has been hypothesized [203]. 

**Figure 11:**
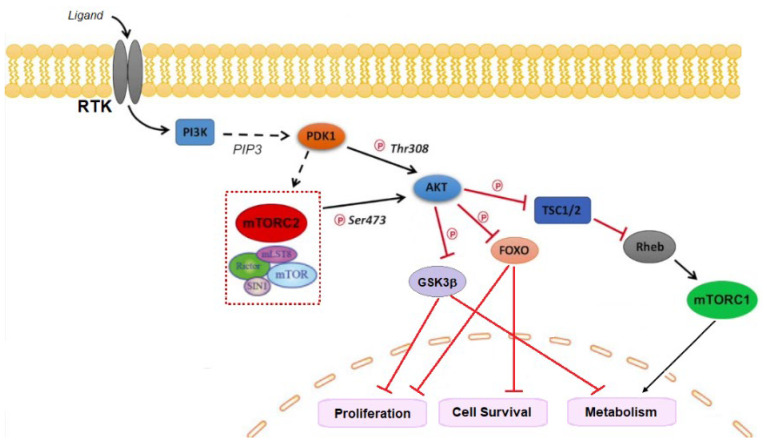
Schematic presentation of AKT1 signaling cascade. Black arrows represent signaling activation while red bars indicate inhibitory signals.

The PI3K signaling pathway is activated as a result of the binding of a growth factor or ligand to a number of membrane-associated receptor tyrosine kinases (RTKs). Activation of RTK leads to the recruitment of the p85 subunit and a subsequent conformational change that allows the p110 subunit to catalyze the conversion of phosphatidyl-inositol-4,5-bisphosphate (PIP2) to the second messenger phosphatidyl-inositol 3,4,5-trisphosphate (PIP3). Tensin homolog deleted on chromosome 10 (*PTEN*) phosphatase, together with the tumor suppressor inositol polyphosphate 4-phosphatase type II (INPP4B), negatively controls PI3K by converting PIP3 to PIP2 thorough dephosphorylation, thus regulating PIP3 levels. PIP3 activates the downstream PI3K pathway by colocalization of the serine/threonine kinases PDK1 and AKT to the cell membrane via their pleckstrin homology (PH) domains. Here, AKT undergoes a double phosphorylation, one on the kinase domain (T308, T309, and T305 for AKT1, 2, and 3, respectively) by PDK1 and another on the regulatory domain (S473, S474, and S472 for AKT1, 2, and 3, respectively) by the mToR complex 2 (mTORC2), resulting in its full activation. Once activated, AKT phosphorylates and inhibits its downstream targets, including tuberos sclerosis complex 2 (TSC2), with the consequent RHEB-GTP accumulation that, in turn, activates mTORC1, glycogen synthase kinase-3β (GSK3β), and the forkhead kinase transcription factors (FOXO). The signaling results in the regulation of cell proliferation, survival, and metabolism (Figure 11).

### 9.1. Ipatasertib (***29***)

**Figure 12:**
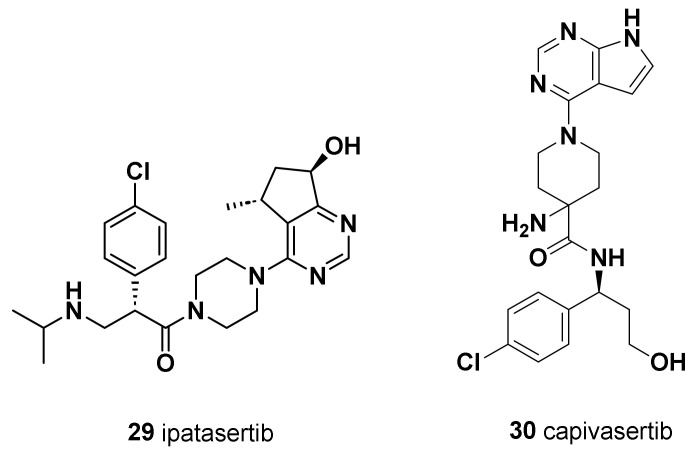
Chemical structure of AKT inhibitors.

Ipatasertib is a selective oral ATP-competitive small-molecule AKT inhibitor with anticancer activity in several cancer types, including prostate, breast, ovarian, colorectal, and non-small-cell lung cancers (Figure 12) [204]. Ipatasertib was the first AKT inhibitor to yield positive outcomes for TNBC treatment. PFS was longer in patients who received ipatasertib than in those who received placebo. The LOTUS trial investigated the addition of ipatasertib to PTX as first-line therapy for TNBC [205]. In PIK3CA/AKT/PTEN-altered tumors, the median progression-free survival of ipatasertib-treated patients was higher compared to the untreated patients, with significant improved survival outcomes. However, a significant pathological complete response rate in early TNBC patients was not observed in the FAIRLANE trial [206].

### 9.2. Capivasertib (AZD5363) (***30***)

Capivasertib is an orally available inhibitor of all three isoforms, AKT1, AKT2, and AKT3, that belongs to the category of ATP-competitive inhibitors (Figure 12) [207]. It acts by preventing substrate phosphorylation by AKT and downregulates the phosphorylation levels of GSK3β and the proline-rich AKT substrate of 40 kDa (PRAS40) biomarkers present in many cancer cells. Capivasertib achieved dose-dependent growth inhibition of xenografts derived from various tumor types, including HER2(+) breast cancer models resistant to trastuzumab. In breast cancer xenografts, capivasertib demonstrated synergistic activity with docetaxel, apatinib, and trastuzumab [208]. Capivasertib showed preclinical activity in TNBC models; the drug sensitivity has been correlated to PI3K or AKT activation and/or PTEN deletion. The drug combination of capivasertib with PTX in TNBC therapy resulted in significantly longer PFS and OS [209]. In preclinical studies, capivasertib, as a single agent in combination with anti-HER2 agents, effectively inhibited the growth of tumors with PIK3CA or MTOR alterations. Phase I/II trials evaluating the co-administration of capivasertib with PTX, with fulvestrant, an estrogen receptor antagonist, or with olaparib in hormone receptor (HR)-positive and HER2-negative breast cancer, demonstrated greater efficacy with an acceptable toxicity profile [210].

## 10. Mammalian Target of Rapamycin (mTOR)

**Figure 13:**
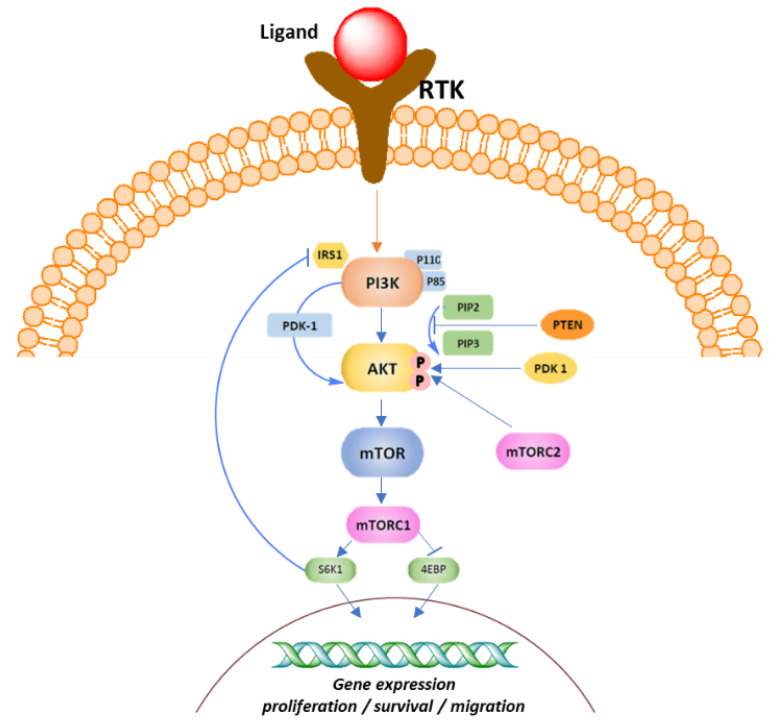
Schematic presentation of PI3K/AKT/mTOR signaling pathway.

mTOR is a serine–threonine kinase activated by both PI3K and MAPK signaling. AKT activation leads to protein synthesis and cell growth by activating the downstream effector mTOR through TSC1/2. Activated mTOR forms two functionally different complexes: mTOR complex mTORC1 and mTORC2 (Figure 13) [211]. mTORC1, on one hand, mediates growth stimulatory effects of mTOR by promoting mRNA translocation and activating protein translation; on the other hand, it is involved in lipid synthesis and metabolism. By contrast, mTORC2 regulates AKT phosphorylation and stimulates further AKT activation and is involved in the organization of the actin cytoskeleton.

### Everolimus (***31***)

**Figure 14:**
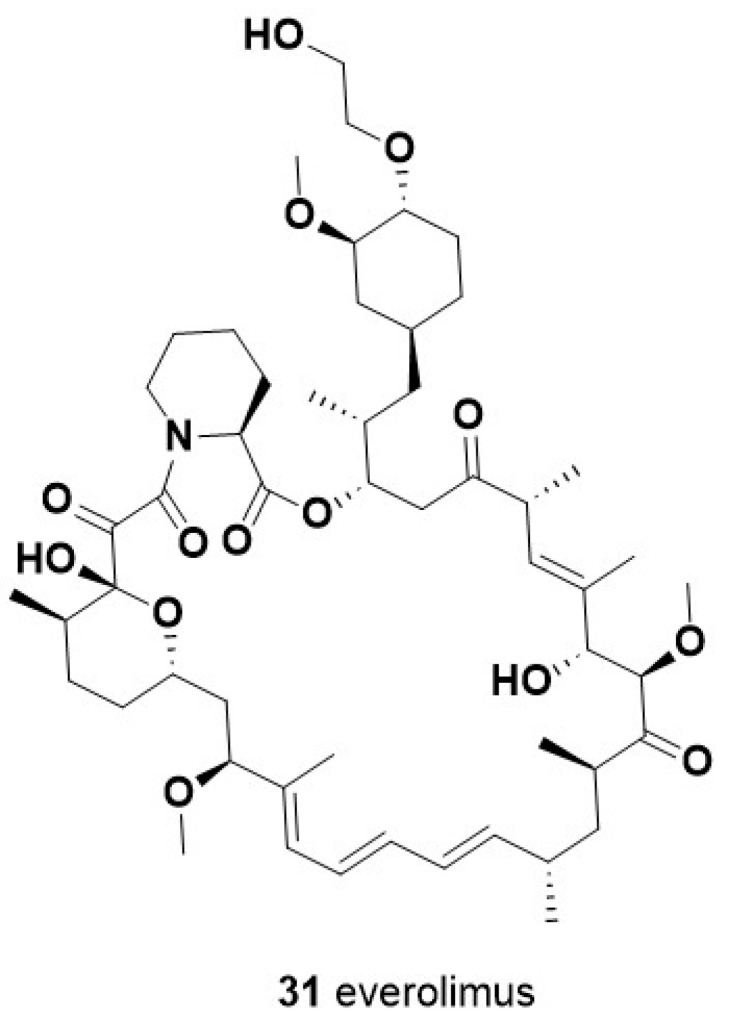
Chemical structure of mTOR inhibitor.

Everolimus is an inhibitor of the serine–threonine kinase mammalian target of rapamycin; it is structurally correlated to the natural macrocyclic lactone sirolimus ((-)-Rapamycin) produced by the bacterium *Streptomyces hygroscopicus* and shows immunosuppressant and anti-angiogenic properties (Figure 14). Several clinical trials have reported the effectiveness of everolimus used in combination with cisplatin in TNBC patients who had residual disease post-standard neoadjuvant chemotherapy. This combination was active in a subset of patients with germline *PALB2* mutation or somatic *PI3KCA* mutation [212].

## 11. Vascular Endothelial Growth Factor Receptor (VEGFR)

The vascular endothelial growth factor (VEGF) family consists of five members: EGF-A, VEGF-B, VEGF-C, VEGF-D, and placenta growth factor. Among them, the most extensively studied is the isoform VEGF-A. The latter executes its function through the binding to VEGF receptor. In contrast to individuals with other BCs, TNBC patients exhibited notably elevated levels of intratumoral VEGF. Moreover, they experienced considerably reduced periods of recurrence-free survival and OS, characterized by a shorter time span between diagnosis and relapse, as well as between relapse and mortality [213]. It is noteworthy that VEGF levels demonstrated a consistent association with unfavorable outcomes, regardless of factors such as tumor size, nodal status, histologic grade, patient age, or the nature of relapse. Therefore, the VEGF pathway has been explored as a valuable therapeutic target in TNBC.

### 11.1. Apatinib (***32***)

**Figure 15:**
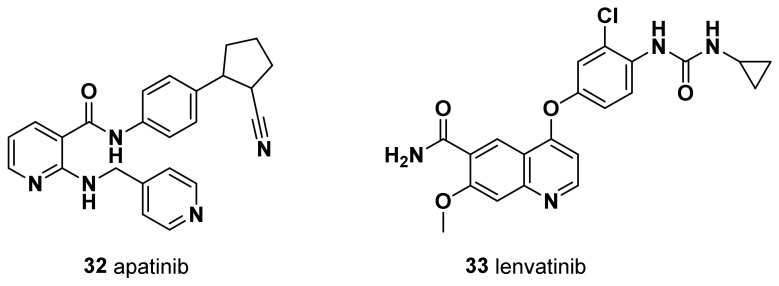
Chemical structure of tyrosine kinase inhibitors.

Apatinib is an orally bioavailable tyrosine kinase inhibitor targeting vascular endothelial growth factor receptor-2 (VEGFR-2), a receptor involved in the growth of blood vessels, with promising anti-angiogenesis and antitumor activity for TNBC [214,215] (Figure 15). In the clinical study NCT01176669, apatinib monotherapy showed promising efficacy in heavily pretreated patients with metastatic TNBC in China [216]. In addition, in another study (NCT03254654), patients with advanced TNBC who did not respond to first/second-line treatments received vinorelbine as monotherapy or vinorelbine with apatinib in 28-day treatment cycles. According to the available data on phase III trials enrolling TNBC patients, the PFS was significantly longer in the group treated with the combination of apatinib and vinorelbine than the vinorelbine group (3.9 months vs. 2.0 months; hazard ratio, 1.82; 95% confidence interval [CI], 1.06 to 3.11; *p* = 0.026) [217]. The combination therapy of apatinib plus etoposide exhibited a promising clinical effect and acceptable toxicity in recurrent or metastatic TNBC as second-or higher-line treatment [187]. The drug combination of apatinib with camrelizumab demonstrated favorable therapeutic effects and a manageable safety profile in patients with advanced TNBC [188].

### 11.2. Lenvatinib (***33***)

Lenvatinib, a small-molecule tyrosine kinase inhibitor that inhibits VEGFR-1 (FLT1), VEGFR-2 (KDR), and VEGFR-3 (FLT4), in combination with an immune checkpoint inhibitor, may have significant clinical activity in selective patients with heavily pretreated metastatic TNBC [218] (Figure 15). In combination with pembrolizumab, lenvatinib showed promising antitumor activity with manageable toxicity in patients with previously treated advanced TNBC [219].

## 12. Epidermal Growth Factor Receptor (EGFR)

The epidermal growth factor receptor (EGFR), a member of the ErbB receptor tyrosine kinase (TK) family, is a transmembrane glycoprotein containing an extracellular ligand binding domain and an intracellular RTK domain, and conveys cell proliferation and differentiation extracellular signals from the outer cell surface to the cell interior [220,221]. When activated by its ligands, such as epidermal growth factor (EGF) and transforming growth factor alpha (TGF-α), EGFR forms dimers with another EGFR, leading to self-phosphorylation and initiating a cascade of intracellular signaling events. These events involve the activation of the Ras/Raf/mitogen-activated protein kinase (MAPK), signal transducer and activator of transcription (STAT), and protein kinase C (PKC) pathways, resulting in cell proliferation and survival, invasion, metastasis, and angiogenesis. Recent studies have shown that a subset of TNBC tumors overexpress EGFR, in particular, the basal subtypes of which EGFR is a characteristic marker [222], making it a potential therapeutic target [223]. Overexpression of EGFR can lead to increased cell proliferation and survival, contributing to cancer growth.

### 12.1. Afatinib (***34***)

**Figure 16:**
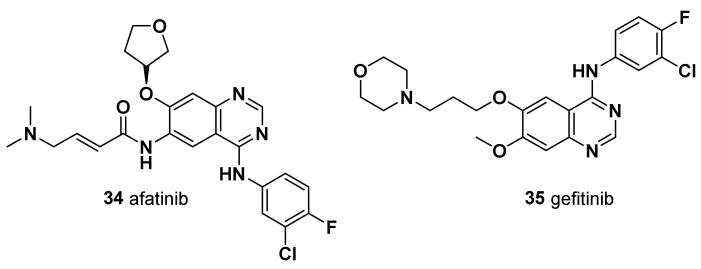
Chemical structure of EGFR inhibitors.

Afatinib is an orally bioavailable EGFR inhibitor with antitumor activity (Figure 16). A study demonstrated that afatinib achieved clinical benefit for at least 4 months in a small number of heavily pretreated unselected patients with TNBC [224]. Preclinical evidence showed that co-administration of EGFR inhibitors may potentiate anti-TNBC chemotherapy [225]. Treatment of metastatic TNBC patients with the anti-EGFR antibody cetuximab plus cisplatin achieved a response superior to the cisplatin monotherapy [226], thus validating EGFR as a target for TNBC [227]. 

The drug combination of afatinib with neoadjuvant PTX achieved a modest effect in TNBC. It was suggested that activation of PI3K and JAK2 pathways and mutation of homologous recombination genes reduced the therapeutic response, while amplification of *DAXX* genes, according to previously reported sensitivity to PTX chemotherapy [228], potentiated the response to afatinib in combination with PTX [229].

### 12.2. Gefitinib (***35***)

The antitumor activity of the EGFR tyrosine kinase inhibitor gefitinib was examined in combination with the mTOR inhibitor everolimus in TNBC cells, with or without activating mutations in the PI3K/AKT/mTOR signaling pathway (Figure 16). The drug combination of gefitinib plus everolimus showed synergistic inhibition in the PI3K and PTEN-mutant CAL-51 cell line but not in the PTEN-null HCC-1937 cell line, with significant effects on cell cycle progression and induction of apoptosis. The results suggest this drug combination as an effective treatment for TNBC with activated mutations of PI3K [230]. 

Recently, Abdelmalek et al., reported the development of a series of hybrid compounds through the combination of tamoxifen, or its active metabolite endoxifen, with the EGFR-inhibitor gefitinib via a covalent linkage [231]. The results revealed that these drug conjugates retained both EGFR inhibition and ER antagonist activities of the parent compounds. Moreover, the most potent analogues showed single digit nanomolar activities at both targets. Two amide-linked endoxifen–gefitinib drug conjugates displayed nanomolar IC_50_ values in the TNBC cells [231].

## 13. SRC

The failure of cancer chemotherapy is due to the presence of cancer stem-like cells, a subpopulation of cancer cells that are able to self-renew and to generate heterogeneous tumors [232]. The aggressive phenotype of TNBC is in part attributed to the presence of intrinsically resistant to chemotherapy breast cancer stem cells (BCSCs). Thus, the priority goal of novel therapies for TNBC therapies is overcoming the BCSC drug resistance. SRC kinase mediates cell proliferation, growth, and survival [233], and its aberrant activation has been observed in breast cancers stemness [234] and metastasis [235].

### Dasatinib (***36***)

**Figure 17:**
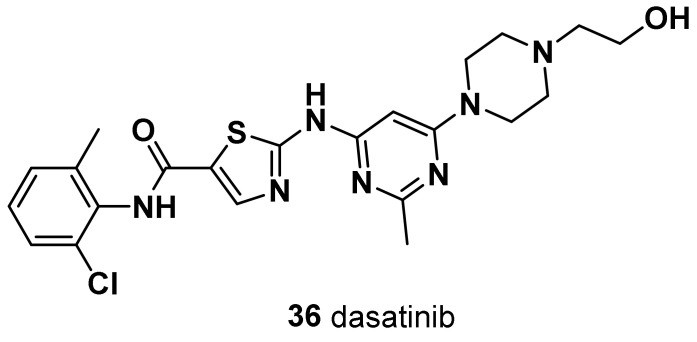
Chemical structure of SRC tyrosine kinase inhibitor.

Dasatinib is a selective SRC tyrosine kinase inhibitor that is used in the therapy of chronic myelogenous leukemia (CML) positive for the Philadelphia chromosome (Figure 17). However, in preclinical studies, dasatinib was shown to inhibit BC cell growth [7]. It has been reported that dasatinib abolished PTX-induced SRC activation as well as BCSC sphere formation capacity and expansion in parental TNBC cells. The drug combination of dasatinib with PTX reduced the drug resistance of PTX-resistant cells in vitro and inhibited breast tumor growth in vivo, suggesting this combination as a potential therapy for overcoming chemotherapy resistance in TNBC patients [236]. 

In addition, a combined treatment with dasatinib and the c-Met inhibitor CpdA (Amgen, Thousand Oaks, CA, USA) significantly inhibited the growth of MDA-MB-231 parental cells and prevented the emergence of dasatinib resistance. The combined treatment with dasatinib and a c-Met inhibitor may block the development of acquired resistance and improve response rates to dasatinib treatment in TNBC [237].

## 14. c-MET

c-MET, also known as hepatocyte growth factor receptor (HGFR), is a protein encoded by the c-MET gene. It was first identified in 1984 by Cooper et al. as a proto-oncogene [238]. C-Met tyrosine kinase signaling cascades are activated via the binding with its single ligand HGF/SF-hepatocyte growth factor/scatter factor and mediate extracellular signals across the cell membrane into cytoplasm. Therefore, c-Met signaling is required for many physiological processes including proliferation, scattering, morphogenesis, and survival. C-Met is overexpressed in 20–30% of all BC cases and in around 52% of TNBC. This observation, together with recent studies on the c-MET signaling pathway, suggests this protein as a novel target to treat TNBC.

### Tivantinib (***37***) 

**Figure 18:**
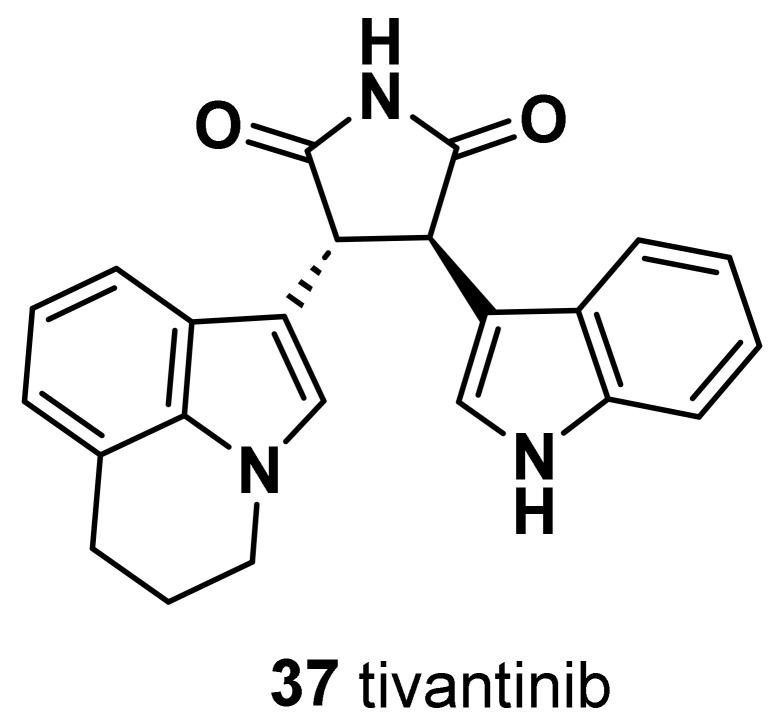
Chemical structure of c-Met receptor tyrosine kinase inhibitor.

Tivantinib is an orally bioavailable inhibitor of c-Met receptor tyrosine kinase (Figure 18). In a phase II trial of patients with metastatic TNBC, tivantinib achieved an overall response rate of 5%, the 6-month PFS rate was 5%, and toxicity was negligible. Even if it was well tolerated, it did not fulfill prespecified statistical targets for efficacy [239]. The sensitivity to c-Met inhibition in TNBC cells was increased upon dasatinib treatment [237].

## 15. Janus Kinase (JAK)

The mammalian JAK-STAT signaling pathway consists of four JK domain-containing proteins, namely, JAK1, JAK2, JAK3, and tyrosine kinase 2 (TYK2), along with seven signal transducers and activators of transcription abbreviated as STATs (STAT1, STAT2, STAT3, STAT4, STAT5A, STAT5B, and STAT6) [240]. Dysregulation of this pathway plays a pivotal role in oncogenic processes, including tumorigenesis, proliferation, angiogenesis, oncogenic signaling, cell survival, anti-apoptosis mechanisms, and immune responses [241].

### Ruxolitinib (***38***) 

**Figure 19:**
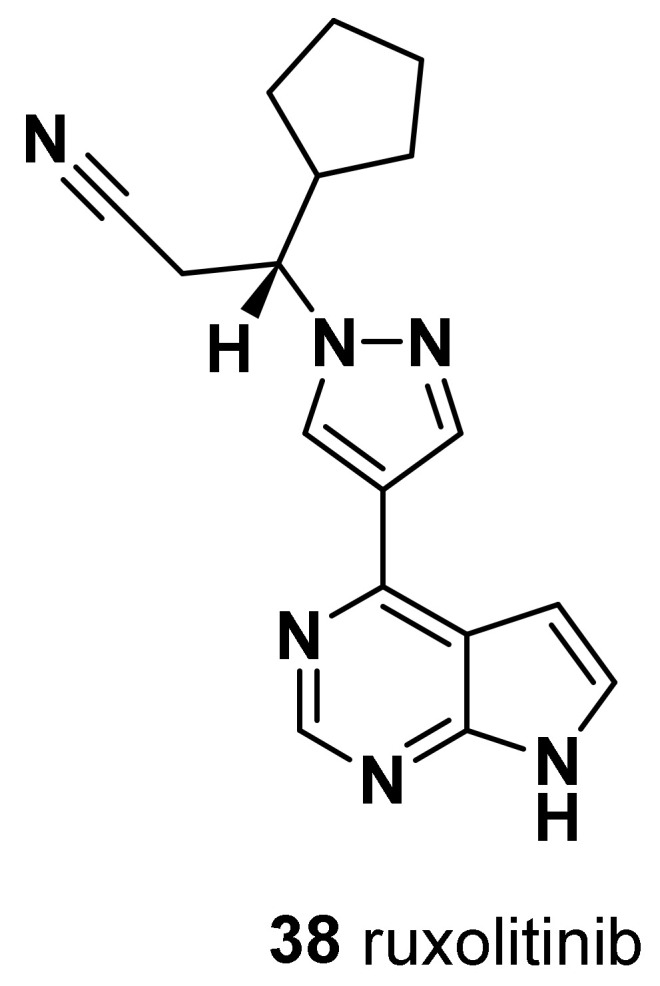
Chemical structure of JAK inhibitor.

Ruxolitinib is an orally bioavailable inhibitor of Janus-associated kinase (JAK) JAK 1 and 2 with antitumor and anti-inflammatory activity (Figure 19). It has been approved for the treatment of patients with myelofibrosis and those with polycythemia [242]. JAK2 signaling was associated with PTX resistance in TNBC patient-derived xenograft models and regulated PTX-resistant cancer-associated fibroblast phenotype transition [243]. In a non-randomized phase II study enrolling pSTAT3-positive patients with refractory, metastatic TNBC, ruxolitinib, as a single agent, was well tolerated but did not meet the primary efficacy endpoint, despite evidence of on-target activity [244]. Novartis has reported that ruxolitinib phosphate is under clinical development and currently in phase II for TNBC. According to GlobalData, phase II drugs for TNBC have a 25% phase transition success rate indication benchmark for progressing into phase III [245].

## 16. Inhibitor of Apoptosis Protein (IAP)—Second Mitochondria-Derived Activator of Caspases (SMAC)

Inhibitors of Apoptosis Protein (IAP) are well known for their antiapoptotic activity. Usually, members of the IAP family are characterized by the presence of the baculoviral IAP repeat (BIR) domain, which engages in physical interactions with caspase proteins, consequently inhibiting the functionality of the latter [246]. Second mitochondria-derived activator of caspases (SMAC) mimetics have arisen as a promising category of targeted therapies currently undergoing clinical evaluation for both solid tumors and hematological cancers. These drugs inhibit the activity of inhibitors of apoptosis proteins; therefore, the dysregulation of IAPs promotes tumorigenesis, metastasis, angiogenesis, and therapeutic resistance, including chemotherapy and radiotherapy [246].

### LCL161 (***39***)

**Figure 20:**
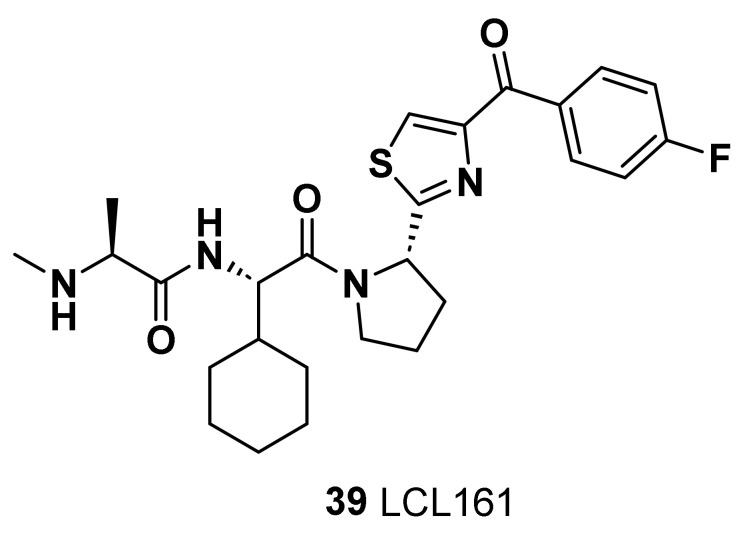
Chemical structure of IAP-SMAC inhibitor.

*LCL161* is an orally bioavailable IAP antagonist and mimics second mitochondria-derived activator of caspases with both pro-apoptotic and antiproliferation effects in cancer cells (Figure 20). At the molecular level, LCL161 binds to the BIR3 domain of cIAP1 and cIAP2 with high affinity and induces cIAP1 and cIAP2 autoubiquitination and proteasome degradation, resulting in the activation of non-canonical NF-κB signaling pathways and the production of tumor necrosis factor α (TNF-α). LCL161 has been shown to induce tumor necrosis factor (TNF)-mediated apoptosis in sensitive cell lines [247], including apoptosis in TNBC xenograft models with TNFα-based gene expression signatures associated with LCL161 [248].

In addition, many preclinical studies have demonstrated the synergistic anticancer activity of LCL161 in combination with different anticancer agents such as PTX, vincristine, and obatoclax. A neoadjuvant trial provided evidence supporting a biomarker-driven targeted therapy approach with LCL161 for selected patients with gene expression signature-positive TNBC [249].

## 17. Splicing Inhibitors

Splicing is the molecular process of introns removal and the joining of exons which mediates maturation of newly transcribed pre-mRNAs into functional mRNA (mRNA). Small molecules inhibiting this process have been shown to exert a potent antitumoral activity, especially in tumors displaying aberrant activity of the oncogenic transcript factor c-MYC [250,251,252,253]. Indeed, the increased activity of this transcription factor dramatically increases the number of synthetized RNA transcripts, creating an overload for the splicing machinery that makes cancer cells vulnerable to its chemical inhibition [254]. In line with the amplification and overexpression of c-MYC which characterizes TNBC, with respect to both normal mammary tissue and other BCs, numerous preclinical studies have demonstrated the antitumoral activity of several splicing inhibitors against cell lines, xenografts, and patient-derived xenograft (PDX) models of this tumoral subtype. The tested inhibitors include a broad list of small molecules acting on different molecular targets. Inhibitors of the SF3b complex, a main spliceosomal component, are the first class of small molecules targeting splicing catalysis to be developed and tested as antitumoral compounds, also in TNBC [250,251]. This class includes sudemycin-D6 [254,255], pladienolide B and its derivatives E7107 [256,257], and H3B-8800, an orally bioavailable analogue of E7107 [255]. These molecules act by interacting with the SF3b complex and preventing U2snRNP interaction with target pre-mRNA, which leads to splicing inhibition and consequent intron retention or exon skipping [250,251]. These agents were found to reduce the growth of TNBC cell lines and xenografts, as well their metastatic spread [254,255,256,257]. Unfortunately, phase I trials testing E7107 in solid tumors revealed severe ocular toxicity, leading to the suspension of the study [258,259]. No similar issues were reported for its derivative H3B-8800 in a trial in leukemia patients [260], which opens up the opportunity for the evaluation of its antitumoral efficacy also for solid tumors, including TNBC.

Another splicing inhibitor with proven efficacy against TNBC preclinical models is the type-I protein arginine methyltransferases (PRMTs) inhibitor MS-023 [261,262]. PRMTs inhibitors interfere with the splicing process by impairing the asymmetric dimethylation of both core spliceosomal proteins and accessory splicing factors [251,263,264]. Similar to the reports for H3B-8800, treatment with MS-023 was shown to induce a global inhibition of the splicing process, which leads to the accumulation of intron retaining transcripts, many of which form cytotoxic double-stranded RNAs (dsRNAs) [255,262]. By mimicking a viral infection, these endogenous dsRNAs activate the cellular antiviral defense pathways, eliciting secretion of inflammatory cytokines and type 1 IFNs and activation of the extrinsic apoptotic pathways [255,262]. Furthermore, at least for H3B-8800, whose efficacy was tested also against TNBC xenografts transplanted in immunocompetent mice, splicing inhibition was shown to enhance the antitumoral activity of the adaptive immune system by promoting the infiltration of cytotoxic CD8^+^T cells [255]. In this regard, since high levels of infiltrating lymphocytes have shown to be predictive markers for a better response of TNBC patients to chemotherapy [265], it would be interesting to test if splicing inhibitors sequentially combined with conventional chemotherapy might increase TNBC chemosensitivity by enhancing their leukocyte infiltration grade. Furthermore, several splicing inhibitors, with different chemistry and target proteins, were shown, in other tumoral contexts, to elicit T-cell-mediated immune responses by promoting the generation of splicing derived neoantigens [266,267]. Whether this occurs also in TNBC is still unexplored, but it is highly probable that such mechanism might concur to the antitumoral activity of this class of inhibitors also in these tumors. MS023 is still at the preclinical developmental stage, while a phase 1 clinical study testing the safety and efficacy of the PRMTs type I inhibitor GSK3368715 in patients with advanced solid tumors was terminated early because of the risk of thromboembolic events exceeding any potential benefit [268]. Further studies on PRMTs inhibitors are thus needed to translate from the bench to the bedside the putative antitumoral effects of this class of splicing inhibitors.

Another class of small molecules targeting the splicing process, showing promising antiviral effects in TNBC preclinical models, but still far from the clinical testing, are splicing factor kinases inhibitors, for which we guide readers to a specific review on the topic [75].

Overall, several preclinical studies have demonstrated the high potential for multiple splicing inhibitors to be further developed in efficacious therapies against TNBC, both as novel chemotherapeutic agents and pro-immune enhancing agents. Furthermore, the multilayered control of the splicing process offers multiple ways for the development of pharmacological strategies to interfere with it.

## 18. Recent Promising Biomarkers as Future Therapeutic Approaches

Despite the recent drugs approved for the treatment of TNBC and used in combination with standard chemotherapy, as previously described in the present review, there is still an urgent need to identify alternative molecular targets, along with novel molecular biomarkers involved in cancer progression, to improve the outcome of chemotherapy and to overcome the insurgence of chemotherapy-resistant TNBC. 

### 18.1. Paraoxonase-2 Enzymes

Within this framework, increasing evidence has recently suggested that the enzyme paraoxonase-2 (PON2) could play a key role in various solid malignancies, enhancing cancer cells’ capacity to mitigate the high levels of oxidative stress encountered by these cells [269]. This, in turn, accelerated their rate of proliferation and ultimately contributed to heightened resistance to different chemotherapeutics (imatinib, doxorubicine, staurosporine, or actinomycin) in cell culture models.

PON2, represents one of three highly conserved members of the *PON* gene family of enzymes consisting of PON1, PON2, and PON3. Their name was inspired by the member PON1, a calcium-dependent esterase that was first described for its capacity to hydrolyze organophosphates and the insecticide parathion, whose active metabolite paraoxon functions as a neurotoxic cholinesterase inhibitor [270]. In humans, PON1 and PON3 are synthesized predominantly in the liver, from where they are secreted into the blood, and are associated with high-density lipoproteins (HDL), whereas human PON2 is ubiquitously expressed in a wide range of tissues and its protein product localizes to the endoplasmic reticulum and nuclear envelope [270,271]. At the plasma membrane level, PON2 is a transmembrane protein with its enzymatic domain facing the extracellular compartment and thus plays an important role in rescuing the peroxidation of membrane components [272,273]. Therefore, it has been hypothesized that PON2 plays a pivotal role in contributing to antioxidant activity against lipoperoxides on HDL particles.

Based on the evidence that, in many types of cancers, PON2 is upregulated in tumor tissues relative to corresponding normal tissues, Campagna et al. firstly evaluated the PON2 levels in BC subtypes and subsequently investigated the role played by the enzyme in TNBC cell metabolism, focusing on the possibility of the enzyme in affecting the tumor cell sensitivity to the chemotherapeutic treatment [274]. The immunohistochemical analysis revealed an upregulation of this enzyme in the infiltrating BC of the subtypes Luminal A, HER2^+^, and TNBC compared to the healthy tissue. Remarkably, the deficiency of PON2 caused apoptosis of the tumor cells, demonstrating an oncogenic function. 

Subsequently, in vitro effects of PON2 downregulation on cell proliferation and response to chemotherapeutics were evaluated clearly highlighting the potential of PON2 to be a promising molecular target capable of improving the efficacy of the chemotherapy in TNBC patients, who, to date, still lack targeted therapies. At present, no PON2 inhibitors have been described in the literature, making the development of efficient drugs a major research challenge. 

### 18.2. Nicotinamide N-Methyltransferase Enzyme

Furthermore, similarly, nicotinamide *N*-methyltransferase (NNMT), another potential prognostic marker for TNBC, has recently been demonstrated to be overexpressed in TNBC tumors and to be associated with the development of resistance [275,276,277,278,279,280]. Indeed, Wang et al., in 2019, reported that NNMT expression in breast cancer inhibited apoptosis and enhanced chemotherapy resistance, resulting in high metastasis and poor survival, and demonstrated that NNMT expression in tumor tissues of TNBC patients was higher than that in other subtypes [281].

NNMT, a phase II metabolizing enzyme, catalyzes the transfer of the methyl units from *S*-adenosyl-l-methionine (SAM) to nicotinamide (NAM), the precursor of a large number of energy metabolism-related molecules, such as NAD^+^ and NADH, producing the stable metabolic products 1-methylnicotinamide (1-MNA) and *S*-adenosyl-L-homocysteine (AdoHcy); this thereby decreases the overall methyl pools for RNA, histone, and DNA methylation and contributes to a wide range of changes in cancer-associated gene expression [282].

A multitude of NNMT-induced mechanisms, including SAM depletion-induced DNA/histone hypomethylation and the associated upregulation of tumor-promoting genes, have been shown to promote tumorigenic phenotypes in various solid tumors including breast cancer [282].

In a more recent study, Wang et al., investigated the function and mechanism of NNMT on the metastasis of TNBC [283]. Through in vitro assays, they found that NNMT overexpression promoted the migration and invasion of TNBCs by enhancing membrane fluidity and epithelial–mesenchymal transition (which is generally considered to be a major driver of metastasis) of TNBCs through the reduction in cholesterol levels in the cytoplasm and cell membrane. Mechanistically, it has been reported that NNMT repressed protein phosphatase 2A activity to activate the MEK/ERK/c-Jun pathway, leading to the increase in ABCA1 expression in TNBC cells and the reduction in cholesterol levels and EMT activation, which finally enhances migration and invasion in vitro and metastasis capacity in vivo.

#### 18.2.1. NNMT Bisubstrate Inhibitors

Highly potent, selectively targeted, and functional NNMT inhibitors are valuable resources for exploring the intricate regulatory mechanisms governed by NNMT and for investigating various pharmacological hypotheses suggesting NNMT as a viable therapeutic target [284]. Despite the growing interest in its implication in many disease states, few cell-active NNMT inhibitors have been described to date, and none have entered clinical trials. At present, the most powerful NNMT inhibitors reported in the literature are compounds developed through a bisubstrate transition state of the methylation reaction mimic approach [285,286]. Basically, these bisubstrate-like inhibitors consist of two covalently connected moieties mimicking the cofactor and substrate, SAM and NAM, respectively.

**Figure 21:**
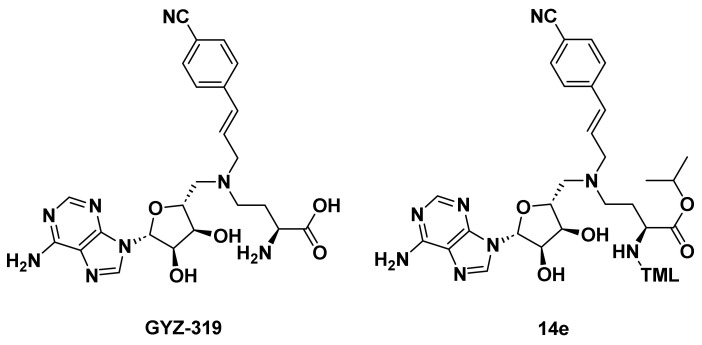
Chemical structure of NNMT bisubstrate inhibitors.

In this regard, Gao et al., designed and developed a series of NNMT inhibitors by introducing a nonbenzamide aromatic moiety substituted with different electron-withdrawing groups to mimic the nicotinamide nucleus, which is connected to the SAM unit through a three carbon trans-alkene linker [287]. Among these compounds, the para-cyano-substituted styrene-based inhibitor GYZ-319 (Figure 21) was identified as the most potent NNMT inhibitor with an IC_50_ value of 3.7 nM. Moreover, ITC experiments confirmed the ability of the inhibitor to strongly bind NNMT, with a dissociation constant of 21 nM. However, the low nanomolar potency exhibited in biochemical assays was not reflected in human cancer cell lines, in which the compound GYZ-319 only showed significant antiproliferative effects at 100 μM. This discrepancy has been explained by the poor cell permeability of GYZ-319, which is due to the presence of two highly polar functional groups present in all potent bisubstrate NNMT inhibitors reported to date.

Therefore, in a subsequent study [288], the authors focused on a prodrug strategy designed to translate the observed potent biochemical inhibitory activity of NNMT inhibitor GYZ-319 into strong cellular activity. The dual prodrug **14e** was thus obtained, bearing the carboxylic acid masked as an isopropyl ester and the amine masked with a trimethyl-lock group in its structure (Figure 21), showing the most promising profile in terms of hydrolytic stability and cellular activity. Indeed, the prodrug exhibited improved cell permeability, which also translated to a significant enhancement of cellular activity compared to the parent compound.

#### 18.2.2. NMMT Macrocyclic Peptides Inhibitors

In another approach, van Haren et al. [289] applied a peptide-mRNA display technology, known as the random nonstandard peptide integrated discovery (RaPID) system, to screen a library of more than 1012 macrocyclic peptides binding to NNMT. A set of macrocyclic peptides were identified with affinity for NNMT. In cell-based assays, administration of the previously synthesized macrocyclic peptides showed a reduction in the concentration of MNA, indicating a target-specific effect. Interestingly, this inhibition was found not to be impacted by elevated concentrations of either NA or SAM substrates, suggesting that these peptides function as allosteric inhibitors, the first to be reported for NNMT. 

Taking these findings together, it appears clear that elevated NNMT levels are typically associated with unfavorable clinical parameters such as tumor size and advanced stage and grade, making NNMT an attractive and viable therapeutic target. Moreover, it has been demonstrated that NNMT gene silence promotes proliferation, migration, and therapy resistance. Multiple research groups are currently working on the discovery of novel inhibitors with high selectivity and efficacy which will contribute to elucidating the potential mechanism of NNMT and developing novel therapeutic strategies. Although at the moment is unlikely to use NNMT inhibitors alone to treat TNBC cancer, in the future, combining them with traditional targeted drugs, including EGFR-TKIs and bevacizumab, can improve prognosis through an optimized combination therapy, as well as reduce the possibility for the development of drug resistance. With the advancement of further studies and clinical trials, it is strongly believed that NNMT inhibitors can provide substantial benefits for patients with TNBC cancer.

## 19. Conclusions

TNBC represents the most aggressive breast cancer subtype characterized by the absence of expression of the estrogen receptor, progesterone receptor, and human epidermal growth factor receptor-2 which are responsible for the failure of common endocrine therapy and chemotherapy. TNBC patients display poorer outcomes and shorter survival compared to other BC subtypes, primarily due to the earlier onset of metastasis and the insurgence of chemoresistance [290,291]. In particular, the poorest prognosis and higher chances of recurrence are shown by the approximate 30% of TNBC patients who do not achieve a pCR to conventional chemotherapy in the neoadjuvant setting [292]. Therefore, the development of a novel, safe, and cost-effective treatment for TNBC is urgently needed. In the present review, we have summarized the current standard therapeutic armamentarium, and we have described novel small molecules and repositioned drugs developed to target the different molecular characteristics of TNBC subtypes. In addition, we have described innovative dual-targeting agents, combinatory treatments, and novel therapeutic strategies in preclinical testing, such as splicing inhibitors, whose further development retains high promises in improving TNBC management.

Intrinsic and acquired chemoresistance represent the major challenges in the current TNBC therapeutic management. Given the high tumoral heterogeneity within the TNBC subtype, several lines of evidence suggest that entry into the clinical practice of molecular stratification of TNBC patients could help defeat intrinsic chemoresistance [293]. Concerning the drugs described in this review, taxanes and mitotic inhibitors were proposed to display higher efficacy in BL1 tumors; PARP inhibitors were candidates for the treatment of BL2 tumors; inhibitors of CDK4/6 and signaling kinases PI3K, AKT, and mTOR were suggested for LAR tumor treatment [293]. Recent results from the FUTURE phase II umbrella clinical trial support the hypothesis that pharmacological treatments matched to TNBC patient molecular subtypes could improve therapeutic responses, as they showed improvements for IM TNBC patients treated with anti-PD1 plus paclitaxel and for basal-like immune suppressed (BLIS) tumors treated with anti-VEGFR therapies [294]. The administration of patient-tailored targeted first-line chemotherapy surely represents a key step forward also for the management of acquired chemoresistance, as it is conceivable that more effective therapies could limit the chances of the evolution/selection of relapsing resistant tumors. Therefore, the improvement of current therapeutic options for TNBC relies on a better molecular characterization of these tumors, which includes, for example, a comparative omics analysis between primary tumors and secondary tumors, shedding light on the molecular determinants of chemoresistance. These studies could unveil actionable vulnerabilities of recurrent and chemoresistant TNBC, paving the way for novel therapies and allowing patients diagnosed with TNBC to be spared from severe adverse side effects of untargeted therapies and have a better outlook on life.

## Figures and Tables

**Table 1:** Biological effects of mitotic kinase inhibitors in TNBC cells.

Targeted Mitotic Kinase	Cmpd	Biological Effects in TNBC Models		Results from Clinical Studies on TNBC Patients or Other Tumors
		**In Vitro**	**In Vivo**	
AURKB	Barasertib (AZD1152)	Reduction in mesenchymal traits [128].	Inhibition of metastatic spreading of TNBC cells in immunodeficient murine models [128].	Not available. Adequate levels of tolerance in patients with other advanced solid tumors, with observation of stable disease [144,145].
NEK2	CMP3A	Induction of mitotic alterations in synergy with PTX [124]. Induction of sensitivity to PTX in resistant cell lines [124].	Enhancing antitumoral effects of PTX in TNBC cells xenografts and PDXs [124].	Not available.
	JH295	Inhibition of cells migratory and invasive properties [146].	Not available.	Not available.
PLK1	Rigosertib (ON-01910)	Reactivation of Erα expression [147].	Reduction in TNBC cell xenograft and PDX growth [147].	Not available. Moderate toxicity in patients with other advanced solid tumors, with observation of stable disease [148,149].
	Volasertib (BI-6727)	Reactivation of Erα expression [147]. Increased DNA damage, mitotic arrest, and cell death [143,147].	Reduction in TNBC cell xenograft and PDX growth [147].	Tolerable toxicity in patients with other advanced solid tumors, either alone or in combination with other chemotherapy, with partial antitumoral effects [150,151,152,153,154].
	BI-2536	Induction of mitotic abnormalities and apoptosis [125,143,155]. Reactivation of Erα expression [147].	Reduced growth of TNBC PDXs and enhanced activity towards doxorubicin and cyclophosphamide antitumoral effects [125].	Not available. Moderate toxicity and antitumoral effects in patients with other advanced solid tumors [156].
	GSK461364	Synergic antiproliferative and pro-apoptotic effects with docetaxel [157].	Not available.	Not available. Mild toxicity and moderate antitumoral effects in patients with other solid tumors [158].
	Onvasertib	Synergic antiproliferative and pro-apoptotic effects with docetaxel [157].	Synergic antitumoral effects with PTX in TNBC cell xenografts [157].	Not available. One ongoing trail testing the safety and effectiveness of its combination with PTX in locally advanced and metastatic TNBC (NCT05383196).
MPS1/ TTK	CFI-402257	Inhibition of cell growth and induction of apoptosis and aneuploidy [159,160].	Antitumoral effects against TNBC cell xenografts either as a single agent or in combination with carboplatin [160].	Not available. Three ongoing trials testing the safety and antitumoral efficacy in different BC subtypes (NCT02792465, NCT03568422, NCT05251714).
	BOS172722	Induction of cell death either as stand-alone treatment or in combination with PTX [161].	Reduction in the growth of TNBC cell xenografts and PDXs and enhanced activity on the antitumoral effects of PTX [161].	Not available. One ongoing trial testing its safety and tolerability, either alone or in combination with PTX, in advanced nonhematologic malignancies (NCT03328494).
	NTRC 0066-0	Reduction in cell growth and induction of mitotic abnormalities [162].	Reduction in TNBC cell xenograft growth as a single agent. Reduction in the growth of spontaneously developing murine tumors after combination with docetaxel [162].	Not available.
	BAY 1217389	Inhibition of cell growth [163].	Reduction in the growth of TNBC cell xenografts and PDXs and enhanced activity on the antitumoral effects of PTX [163].	Considerable toxicity, without a therapeutic window, in association with PTX [164].
WEE1 kinase	Adavosertib (AZD1775)	Reduced cell growth both as a single agent and in association with capecitabine/5FU and/or ATR inhibitor [165,166,167].	Reduced growth of TNBC xenografts and PDXs both as a single agent and in association with capecitabine/5FU and/or ATR inhibitor [165,166,167].	Adequate levels of tolerance in TNB and other solid tumors, with stable disease in TNBC patients [168]. Not significant antitumoral effects in combination with cisplatin in metastatic TNBC [169].

## Data Availability

Data are contained within the article.

## Abbreviations

5-FU	5-fluorouracil
AR	androgen receptor
BC	breast cancer
BL1	basal-like 1
BL2	basal-like 2
BRCA	breast cancer susceptibility gene
CDK4/6 inhibitors	cyclin-dependent kinase 4/6 inhibitors
CDKs	cyclin-dependent kinases
EGF	epidermal growth factor
ER	estrogen receptor
FDA	Food and Drug Administration
FoxO	forkhead box class O
gBRCA	germline BRCA
GSK3β	glycogen synthase kinase-3β
HER2	human epidermal growth factor receptor 2
HER3	human epidermal growth factor receptor 3
HR	hormone receptor
IAP	inhibitor of apoptosis protein
IC_50_	half-maximal inhibitory concentration
LAR	luminal androgen receptor
M	mesenchymal (M)
mTOR	mammalian target of rapamycin
NAM	nicotinamide
NNMT	nicotinamide *N*-methyltransferase
ORR	objective response rate
OS	overall survival
PARP	poly adenosine diphosphate-ribose polymerase
pCR	pathological complete response
PDXs	patient-derived xenografts
PFS	progression free survival
PI3K	phosphatidylinositol-3-kinase
PKA/B/C	protein kinase A/B/C
PON2	paraoxonase-2
PR	progesterone receptor (PR)
PRAS40	proline rich AKT substrate of 40 kDa
PRMTs	type-I protein arginine methyltransferases
PTEN	phosphatase and tensin homolog
PTX	paclitaxel
RTK	receptor tyrosine kinase
SAM	S-adenosyl-l-methionine
SMAC	second mitochondrial-derived activator of caspases
TNBC	triple-negative breast cancer
TSC2	tuberos sclerosis complex 2
ULK1	Unc-51-like autophagy activating kinase 1
VEGF	vascular endothelial growth factor
VEGFA	vascular endothelial growth factor A
VEGFR	vascular endothelial growth factor receptor
